# Prevalence of Autism Spectrum Disorder and Co-morbidities in Children and Adolescents: A Systematic Literature Review

**DOI:** 10.3389/fpsyt.2021.744709

**Published:** 2021-10-27

**Authors:** Clémence Bougeard, Françoise Picarel-Blanchot, Ramona Schmid, Rosanne Campbell, Jan Buitelaar

**Affiliations:** ^1^Syneos Health, Value Access & HEOR, Montrouge, France; ^2^Servier Global Medical and Patients Affairs, Suresnes, France; ^3^Servier, Global Value & Access, Suresnes, France; ^4^Syneos Health, Value Access & HEOR, London, United Kingdom; ^5^Department of Cognitive Neuroscience, Donders Institute for Brain, Cognition and Behaviour, Radboud University Medical Centre, Nijmegen, Netherlands

**Keywords:** Autism Spectrum Disorder, prevalence, co-morbidities, review, pediatric, autism

## Abstract

**Objective:** Individuals with autism spectrum disorder often present somatic and/or psychiatric co-morbid disorders. The DSM-5 allows for consideration of additional diagnoses besides ASD and may have impacted the prevalence of co-morbidities as well as being limited in capturing the true differences in prevalence observed between males and females. We describe the prevalence of ASD and frequently observed co-morbidities in children and adolescents (<18 years) in the United States and five European countries.

**Methods:** Two systematic literature reviews were conducted in PubMed and Embase for the period 2014–2019 and focusing on the prevalence of ASD and nine co-morbidities of interest based on their frequency and/or severity: Attention Deficit Hyperactivity Disorder (ADHD), anxiety, depressive disorders, epilepsy, intellectual disability (ID), sleep disorders, sight/hearing impairment/loss, and gastro-intestinal syndromes (GI).

**Results:** Thirteen studies on prevalence of ASD and 33 on prevalence of co-morbidities were included. Prevalence of ASD was 1.70 and 1.85% in US children aged 4 and 8 years respectively, while prevalence in Europe ranged between 0.38 and 1.55%. Additionally, current evidence is supportive of a global increase in ASD prevalence over the past years. Substantial heterogeneity in prevalence of co-morbidities was observed: ADHD (0.00–86.00%), anxiety (0.00–82.20%), depressive disorders (0.00–74.80%), epilepsy (2.80–77.50%), ID (0.00–91.70%), sleep disorders (2.08–72.50%), sight/hearing impairment/loss (0.00–14.90%/0.00–4.90%), and GI syndromes (0.00–67.80%). Studies were heterogeneous in terms of design and method to estimate prevalence. Gender appears to represent a risk factor for co-morbid ADHD (higher in males) and epilepsy/seizure (higher in females) while age is also associated with ADHD and anxiety (increasing until adolescence).

**Conclusion:** Our results provide a descriptive review of the prevalence of ASD and its co-morbidities in children and adolescents. These insights can be valuable for clinicians and parents/guardians of autistic children. Prevalence of ASD has increased over time while co-morbidities bring additional heterogeneity to the clinical presentation, which further advocates for personalized approaches to treatment and support. Having a clear understanding of the prevalence of ASD and its co-morbidities is important to raise awareness among stakeholders.

## Introduction

Autism Spectrum Disorder (1) is defined as a lifelong neurodevelopmental disorder characterized by two key symptoms: persistent deficits in social communication/interaction and restricted, repetitive patterns of behavior and abnormal sensory responses (2). The severity of these symptoms varies extensively from one patient to another, leading to a multitude of clinical presentations. Onset of ASD can usually be observed during childhood, with signs detectable as early as 18 months of age (3, 4). However ASD remains extremely challenging to diagnose due to the diversity of clinical presentations and diagnosis requires both awareness from parents and caregivers to detect signs and an assessment from a multidisciplinary medical/paramedical team to confirm signs (4). As a consequence, estimating the prevalence of ASD is challenging, as illustrated by Chiarotti and Venerosi (5) who recently conducted a worldwide narrative review on this topic (except for the African and Latin American regions) and confirmed the high variability in reported prevalence across regions. The authors reported high inter and intra variability across regions, with estimates ranging from 0.42 and 3.13% in Europe, 0.11 and 1.53% in Middle-East, 0.08 and 9.3% in Asia, and 0.87 and 1.85% in North America (5).

Furthermore, individuals with ASD often present co-morbid psychiatric disorders. In a recent review, Hossain et al. (6) reported two studies estimating the prevalence of at least one comorbid psychiatric disorder at 54.8% and up to 94%, with Attention Deficit Hyperactivity Disorder (ADHD), anxiety, depressive disorders and sleep disorders being the most frequent co-morbidities. In addition, individuals with ASD are more likely to experience somatic co-morbidities such as epilepsy, gastro-intestinal (GI) disorders or sight/hearing impairments (7). Both psychiatric and somatic comorbidities further complicate the diagnosis of ASD as they can either exacerbate or mitigate typical symptoms of autism. This can lead to misdiagnosis with inadequate management or delays in diagnosis with missed opportunity for treatment (8). Additionally, numerous studies have demonstrated the negative impacts of co-morbidities on autistic individuals as well as their surroundings, both in terms of quality of life and economic burden (9, 10). The latest version of the Diagnostic and Statistical Manual of Mental Disorders (DSM-5) now allows for consideration of additional diagnoses besides ASD and may have impacted the prevalence of co-morbidities (2, 11). Additionally, the new classification may also have contributed to the limitation of capturing the differences in prevalence observed between males and females for ASD and co-morbidities (7). Ratto et al. (12) provided evidence of potential sex differences in autistic traits and adaptive skills; however, further investigations on the clinical presentations of ASD in males and females are needed to understand the factors explaining these differences in diagnosis rates. As such, it is of importance to have a clear understanding of the prevalence of ASD and its co-morbidities to raise awareness among stakeholders so both can be identified sooner and managed optimally. The objective of this study was to describe the prevalence and time trends of ASD, as well as frequently observed co-morbidities (psychiatric and somatic) in children and adolescents in the United States (US) and five European countries (France, Germany, Italy, Spain, and the UK). As a secondary objective, this study also looked at how age and gender were linked to the prevalence estimates of ASD and its co-morbidities.

## Materials and Methods

A systematic literature review (SLR) was conducted to evaluate the prevalence of ASD in children and adolescents (from 2 to <18 years old) in EU-4 (France, Germany, Spain, and Italy) plus the UK, and the US. This manuscript presents studies reporting the latest prevalence data per country in order to understand the current epidemiological situation of ASD, as well as studies examining the prevalence of ASD overtime. Studies reporting on the prevalence of co-morbidities in ASD were retrieved from a second SLR on the clinical burden of ASD which considered the same population and geographic scope.

### Prevalence of ASD

#### Study Selection

A SLR was conducted on July 24th 2019 in PubMed and Embase for the period 2014–2019, and completed with a gray literature search in December 2019 focused on governmental institutions and research associations such as ASDEU (Autism Spectrum Disorders in the European Union). The search string included the following search terms: “ASD”; “epidemiology” [and related terms]; as MeSH or Emtree terms; or in the title and abstract of articles. The full search strategy for this SLR is presented in Supplementary Material 1.

The search focused on retrieving the latest prevalence estimate per country as well as prevalence over time. Study designs were limited to observational studies. Studies with prevalence as primary outcome were included, regardless of the method. Only articles published in English language were considered for review. Table 1 summarizes the Population, Intervention, Comparator, Outcome and Study Design criteria selected for this review.

**Table 1:** PICOS criteria.

	**Prevalence of ASD**	**Prevalence of co-morbidities**
Population	Pediatric population (from 2 to <18y) with ASD according to DSM-IV/DSM-5	Pediatric population (from 2 to <18y) with ASD according to DSM-IV/DSM-5
Intervention	Not applicable	Not applicable
Comparator	Not applicable	Not applicable
Outcomes	• Prevalence • Trend overtime	• Prevalence for the following co-morbidities: ADHD, anxiety, depressive disorders, epilepsy, ID, sleep disorders, sight/hearing impairment/loss, GI,
Study designs	Observational studies	Observational studies

### Prevalence of Co-morbidities in ASD

#### Search Strategy

The second SLR was conducted on July 24th 2019 in PubMed and Embase for the period 2014–2019. This SLR included several search strings related to the clinical burden of ASD to inform several topics such as risk factors, mortality and prevalence of co-morbidities. The search string included the following search terms: “ASD” [and related terms]; “co-morbidity”; “mortality”; and “risk factor”; as MeSH or Emtree terms; or in the title and abstract of articles. The full search strategy for this SLR is presented in Supplementary Material 2. Only results retrieved from the search string on comorbidity are presented in this paper.

#### Study Selection Criteria

This SLR focused on nine co-morbidities of interest: ADHD, anxiety, depressive disorders, epilepsy, Intellectual Disability (ID), sleep disorders, sight/hearing impairment/loss and GI problems (13). These co-morbidities were selected as they are frequently associated with ASD in our population of interest and correlated with substantial impairment. The selection of co-morbidities was then submitted to clinical experts for approval. A broad definition was considered for each co-morbidity as high heterogeneity in the reporting of outcomes was expected. For example, information on comorbid GI syndromes and hearing impairment was mostly based on reported complaints rather than on medically established diagnoses. Studies reporting on prevalence data for these co-morbidities as primary or secondary outcome were included. Relevant studies from the first search (prevalence of ASD) reporting prevalence of co-morbidities were also included. Geographical scope and population of interest were the same as those stated earlier, and only observational studies were considered. Table 1 summarizes the PICOS criteria established for this review.

#### Study Review Process

Two researchers independently screened the titles and abstracts of identified studies and then full texts, to assess eligibility. Disagreements were resolved by discussion between the two reviewers and decision was made by a third reviewer if no agreement could be found. Information from included articles was extracted into a predefined data extraction template which included study characteristics, target population details and study outcomes.

Due to the variety of different study types included and the challenges in comparing study quality, studies were included without assessment of their methodological quality. As the study design, participant intervention and reported outcomes measured varied markedly, this research focused on describing the studies, their results, their applicability and their limitations and on qualitative synthesis.

## Results

### Prevalence of ASD

We retrieved 13 publications reporting on the latest prevalence data for each country (8 studies) at the time the study was conducted and the evolution of ASD prevalence overtime (6 studies). The PRISMA diagram for this review is presented in Supplementary Material 3.

**Figure 1:**
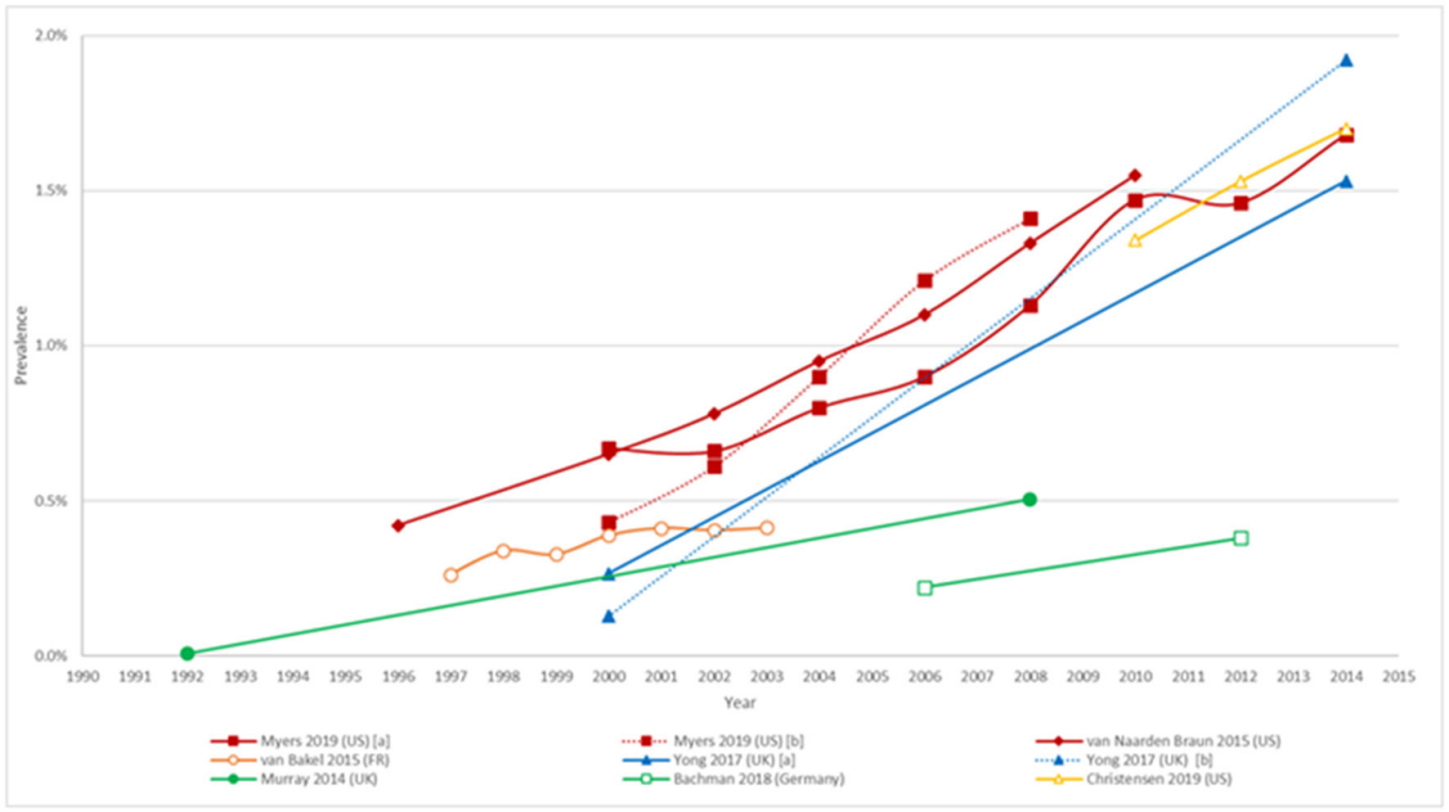
Trends in prevalence of ASD. Myers et al. (21) [a] and [b] include the results from the CDC data set and clinically diagnosed data set, respectively; Yong et al. (22) [a] and [b] include results for the age group 5 to 12 and 13 to 17, respectively. Age of the population considered: 4 year-olds: Christensen et al. (15); 7 year-olds: van Bakel (23); 8 year-olds: Myers et al. (21); van Naarden Braun (24); 5-17 year-olds: Yong et al. (22); 0-24 year-olds: Murray (25), Bachman et al. (20).

Prevalence point estimates were found for each country within our scope and ranged from 0.38% in Germany to 1.85% in the US. Table 2 presents the latest estimates found for each country. All identified studies differed in terms of methodology with differences in age groups, geographical scopes and methods to estimate prevalence. Prevalence trends are summarized in Figure 1. Time trends by gender were found in Bachman et al. (20) and van Naarden et al. (24). No time trends were found for Italy or Spain.

**Table 2:** Latest data on the prevalence of ASD^*^.

**Author (15)**	**Country**	**Year of estimate**	**Setting**	**Method used to estimate prevalence**	**Age group**	**Cohort size (N)**	**Prevalence**		**M/F ratio**
CDC (14)	US	2016	National	Case enumeration and record review	8y	275,419	Total: 1.85% (CI_95%_ 1.80–1.91%) Male: 2.97% (CI_95%_ 2.88–3.06%) Female: 0.69% (CI_95%_ 0.65–0.74%)	1 in 54 children	4.3 CI_95%_ (4.0–4.6)
Christensen et al. (15)	US	2014	National	Case enumeration and record review	4y	70,887	Total: 1.70% (CI_95%_ 1.61–1.80%)	1 in 59 children	NR
ASDEU (1)	Europe	2015	International	TNF, SCQ and national registries	7–9y	631,619	1.22%	1 in 89 children	NR
Delobel-Ayoub et al. (16)	France (South-East)	2015	Regional	Regional registries	7–9y	32,342	Total: 0.48% (CI_95%_ 0.40–0.56%)	1 in 137 children	4.0
	France (South-West)	2015	Regional	Regional registries	7–9y	15,836	Total: 0.73% (CI_95%_ 0.60–0.87%)	1 in 208 children	5.4
Narzisi et al. (17)	Italy	2016	Regional	Disability certificate census + SCQ & TNF	7–9y	10,138	Total: 0.80% (CI_95%_ 0.62–0.97%)	1 in 126 children	5.2
Morales-Hidalgo et al. (18)	Spain	NR	Regional	Screening + Clinical assessment	3–5y	2,755	Total: 1.55% (CI_95%_ 0.89–2.20%) Male: 2.52% (CI_95%_ 1.34–3.71%) Female: 0.58% (CI_95%_ 0.01–1.16%)	1 in 65 children	4.3
	Spain	NR	Regional	Screening + Clinical assessment	10–12y	2,827	Total: 1.00% (CI_95%_ 0.48–1.51%) Male: 1.72% (CI_95%_ 0.71–2.73%) Female: 0.39% (CI_95%_ 0.05–0.83%)	1 in 100 children	4.4
NHS Digital (19)	UK	2017	National	Parent report and direct interviews with children	2–4y	1,463	Total: 1.4% (CI_95%_ 0.70–1.80%) Male: 6.8% Female: 4.2%	1 in 71 children	4.4
	UK	2017	National	Parent report and direct interviews with children	5–19y	7,654	Total: 1.2% (CI_95%_ 0.90–1.40%) Male: 1.9% Female: 0.4%	1 in 83 children	4.8
	UK	2017	National	Parent report and direct interviews with children	5–10y	3,597	Total: 1.5% Male: 2.5% Female: 0.4%	1 in 67 children	6.5
	UK	2017	National	Parent report and direct interviews with children	11–16y	3,121	Total: 1.2% Male: 1.8% Female: 0.7%	1 in 83 children	2.7
	UK	2017	National	Parent report and direct interviews with children	17–19y	936	Total: 0.5% Male: 1.0% Female: N/A	1 in 200 children	NR
Bachmann et al. (20)	Germany	2012	National	Nationwide health insurance database	0–24y	6,400,000	Total: 0.38% Male: 0.53% Female: 0.20%	1 in 264 children	2.7

*ASDEU, Autism Spectrum Disorder in the European Union; CDC, Centers for Disease Control; CI, Confidence Interval; NHS, National Health Service; NR, Not Reported; SCQ, Social Communication Questionnaire; TNF, Teacher Nomination Form*.

* *As of 2019 when the review was conducted*.

In the US, two studies were identified which reported prevalence estimates of 1.70% (CI_95%_: 1.61–1.80%) in children aged four year old (15), and 1.85% (CI_95%_: 1.80–1.91%) in children aged eight year old (26). Additionally the eight year old cohort had a four times higher prevalence rate in males compared to females (2.97 vs. 0.69%). Both studies reported data from the Autism and Developmental Disabilities Monitoring network (ADDM), which uses a multiple-source, records-based surveillance method with 13 sites spread across the country and constitutes an active surveillance system. Case ascertainment involves a two-phase process where records from multiple data sources in the community are reviewed and then assessed by trained personnel to determine ASD case status. The ADDM network has reported ASD prevalence in multiple communities every two year since 2000, thus allowing to follow the evolution of the prevalence over time: Christensen et al. (15) reported prevalence rates in children aged four year of 1.34% (CI_95%_: 1.25–1.44%), 1.53% (CI_95%_: 1.43–1.63%) and 1.70% (CI_95%_: 1.61–1.80%), for the years 2010, 2012, and 2014 respectively. Prevalence over time in children aged eight year old was also found in Myers et al. (21) who reported a steady increase in prevalence from 0.43% (CI_95%_: 0.32–0.54%) in 2000 to 1.41% (CI_95%_: 1.09–1.82%) in 2008, which was in line with the estimates from the CDC who reported estimates of 0.67% (CI_95%_: 0.45–0.99%) and 1.13% (CI_95%_: 0.48–2.12%) for the same years (14). Nevison et al. (27) compared time trends in prevalence in three large US databases: the Individuals with Disabilities Education Act, ADDM Network, and California Department of Developmental Services. The Individuals with Disabilities Education Act database collected data across 50 states in a cohort aged 5–17 year and the California Department of Developmental Services collected data for all individuals living in California meeting the DSM diagnostic criteria for autism. The California Department of Developmental Services was the longest running of the three datasets, reporting an increase in ASD prevalence from 0.001% in the cohort born in 1931 to 1.2% among the 5 year-olds born in 2012. All three datasets displayed a consistent upward trend over time (27).

Prevalence estimates for France and Italy were found in Delobel-Ayoub et al. (16) and Narzisi et al. (17), respectively. Both studies were part of the ASDEU project involving 14 European countries and which aimed at estimating the prevalence of ASD in children aged between 7 and 9 year in 2015. The study by Delobel-Ayoub et al. (16) involved two regional childhood disabilities registries covering the South-West and South-East regions of France (16). Both registries routinely included children aged eight year old, and cases of ASD were ascertained based on review of medical records from hospitals, psychiatric services and autism referral centers. The authors reported prevalence estimates of 0.48% (CI_95%_: 0.40–0.56%) and 0.73% (CI_95%_: 0.60–0.87%) for the South-East and South-West region respectively, however the study mentions that 5% of the parents of potential ASD cases refused to participate which slightly impacts the previous estimates. The South-West cohort also reported a higher male/female (M/F) ratio compared to South-East region (5.4 vs. 4.0). In Italy, Narzisi et al. (17) reported prevalence for the metropolitan area of Pisa (Tuscany, Central Italy). The study considered children aged between 7 and 9 years old and followed a two-step process: (1) identification of certified children with a diagnosis of ASD and (2) identification of new cases. Case ascertainment in the first phase was based on the review of medical records by both the ASDEU team and the local child neuropsychiatry service team. The second phase of the study involved the participation of local school teachers who were asked to fill the Teacher Nomination Form (TNF) and distribute the Social Communication Questionnaire (SCQ) to the parents of children identified with the TNF. Children who had a positive TNF and SCQ (≥9) were administered the Autism Diagnostic-Revised (ADI-R) in association with the Autism Diagnostic Observation Schedule-2 (ADOS-2). Prevalence of ASD following the first phase of the study was 0.8% (N = 81/10, 138) (CI_95%_: 0.62–0.97%) or 1 in 126 children. Prevalence for the second phase of the study was 0.2% (CI_95%_: 0.06–0.33%) and 0.3% (CI_95%_: 0.12–0.45%) when adjusting for non-response. Additionally, males had a five time higher prevalence of ASD compared to females. Prevalence over time for France was reported in van Bakel et al. (23) whose study was based on the same two regional registers considered in Delobel-Ayoub et al. (23) and presented an increase in prevalence, from 0.26% (CI_95%_: 0.22–0.32%) in 1997 to 0.41% in 2003 (CI_95%_: 0.36–0.48%).

A similar study was conducted by Morales-Hidalgo et al. (18) in Spain. The study aimed at estimating the prevalence of ASD, ADHD, and social communication disorder in children aged 4–5 years and 10–11 years in Tarragona (Catalonia, Spain). The research was part of the neurodevelopmental Disorders Epidemiological Research Project and consisted in a screening phase where parents and teachers were administered the Childhood Autism Spectrum Test and the EDUTEA questionnaire respectively. In a second phase, screening was confirmed by interviewing parents of positively screened children using the ADI-R (Autism Diagnostic Interview-Revised) and administering children the ADOS-2 (Autism Diagnostic Observation Schedule) and Wechsler scales. Adjusted prevalence reported by the authors was 1.55% (CI_95%_: 0.89–2.20%) in children aged 4–5 years and 1.00% (CI_95%_: 0.48–1.51%) in children aged 10–11 years, while direct prevalence was 1.06% (CI_95%_: 0.66–1.46%) and 0.78% (CI_95%_: 0.44–1.12%) respectively. Additionally, the authors mentioned that between 1.84 and 2.59% of the children exhibited subclinical diagnoses of ASD. Finally, in both cohorts the prevalence was four times higher in males compared to females.

Prevalence rates for the UK were obtained from the Survey of the mental health of children and young people 2017 available on National Health Service digital which aimed at assessing the prevalence of mental health conditions (including ASD) in children and young people aged 2 to 19 years (19). The method of the survey involved the administration of the Development and Well-Being Assessment questionnaire (28). Case definition for ASD was based on the ICD-10 classification (International Classification of Diseases, Tenth Revision, Clinical Modification) and encompassed the following codes: F84.0 (childhood autism), F84.1 (atypical autism), F84.5 (Asperger syndrome) and F84.8 (other pervasive developmental disorders). Prevalence over time was reported in Yong et al. (22) who analyzed time trends in ASD using the Clinical Practice Research Datalink, an electronic medical records database. The authors observed a steady increase in prevalence in children aged 5–12 and 13–17 years old between the years 2000 and 2014, progressing from 0.27 to 1.53% and 0.13 to 1.92% respectively. The M/F prevalence ratio was substantially higher in the 5–10 year old cohort (M/F ratio = 6.5; male: 2.5%, female: 0.4%) compared to the 11–16 year old cohort (M/F ratio = 2.7; male: 1.8%; female: 0.7%) (22).

Bachmann and colleagues estimated the prevalence of ASD in Germany by conducting a claims database analysis using the Allgemeine Ortskrankenkassen database for the period 2006–2012 (20). Case definition was based on the ICD-10 classifications and involved the following codes: F84.0, F84.1, F84.5, F84.8 and F84.9 (pervasive developmental disorder, unspecified). Bachmann reported an increased prevalence of 0.22 and 0.38% in individuals aged 0 to 24 years for the years 2006 and 2012 respectively. The prevalence increased over time and peaked at 0.60% (males = 0.90%; females = 0.29%) in 6 to 11 year olds. Finally, the M/F ratio (2.7) was the lowest compared to all other countries assessed.

### Prevalence of Co-morbidities in ASD

**Figure 2:**
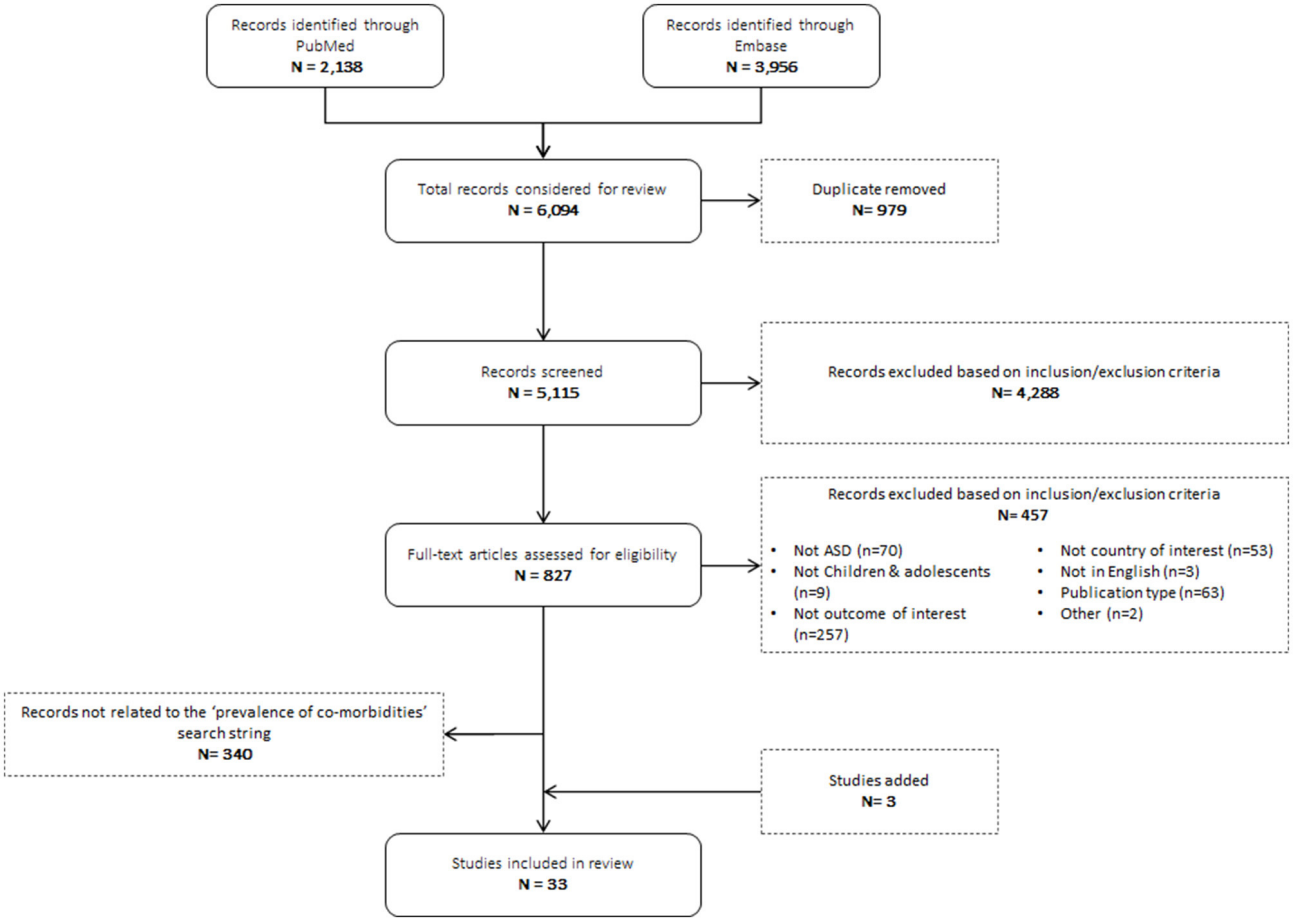
PRISMA diagram prevalence of co-morbidities in ASD.

A total of 6,094 studies were identified. After reviewing titles and abstracts, 827 full text articles were reviewed for relevance. The 340 articles related to mortality and risk factors were not included in this review. 30 studies met the inclusion criteria and 3 studies from the search on prevalence of ASD were also added as they reported prevalence on co-morbidities of interest. Ultimately, 33 studies were included in this review. A PRISMA diagram for this search is displayed in Figure 2. Studies that did not provide point prevalence estimates for co-morbidities were excluded.

Of the 33 studies included, 26 (79%) were conducted in the US while seven were conducted in a European country (France [*n* = 2], Italy [*n* = 2], UK [*n* = 3]). Additionally, prevalence of co-morbidities was reported as a primary outcome in 19 (58%) studies and as a secondary outcome in the other 14 publications. Studies were heterogeneous in terms of study design, with a mix of cohort studies (*n* = 23), cross sectional studies (*n* = 4) and case-control studies (*n* = 6). Studies were also heterogeneous regarding the method considered to estimate the prevalence of co-morbidities: tools and scales (± combined with clinical assessment) were used in 18 studies while eight considered database or registries and seven relied on clinical expertise or caregivers report only. All studies considered cohorts with more than 30 autistic individuals, the lowest sample size being retrieved in Mosner et al. (29) with 35 adolescents. In addition, case ascertainment was based on self-reporting in four studies (30–33) and sample characteristics were missing in Polyak et al. (34).

The number of studies per co-morbidity was as follows: ADHD (*n* = 17), anxiety (*n* = 13), depressive disorders (*n* = 12), epilepsy/seizure (*n* = 12), GI syndromes (*n* = 7), hearing impairment (*n* = 3), ID (*n* = 13), sleep disorder (*n* = 7), and vision impairment (*n* = 3). Study characteristics are presented in Supplementary Material 4, and a summary of results stratified by age (0–5y; 6–11y and 12–17y) can be found in Supplementary Material 5.

#### ADHD

**Figure 3:**
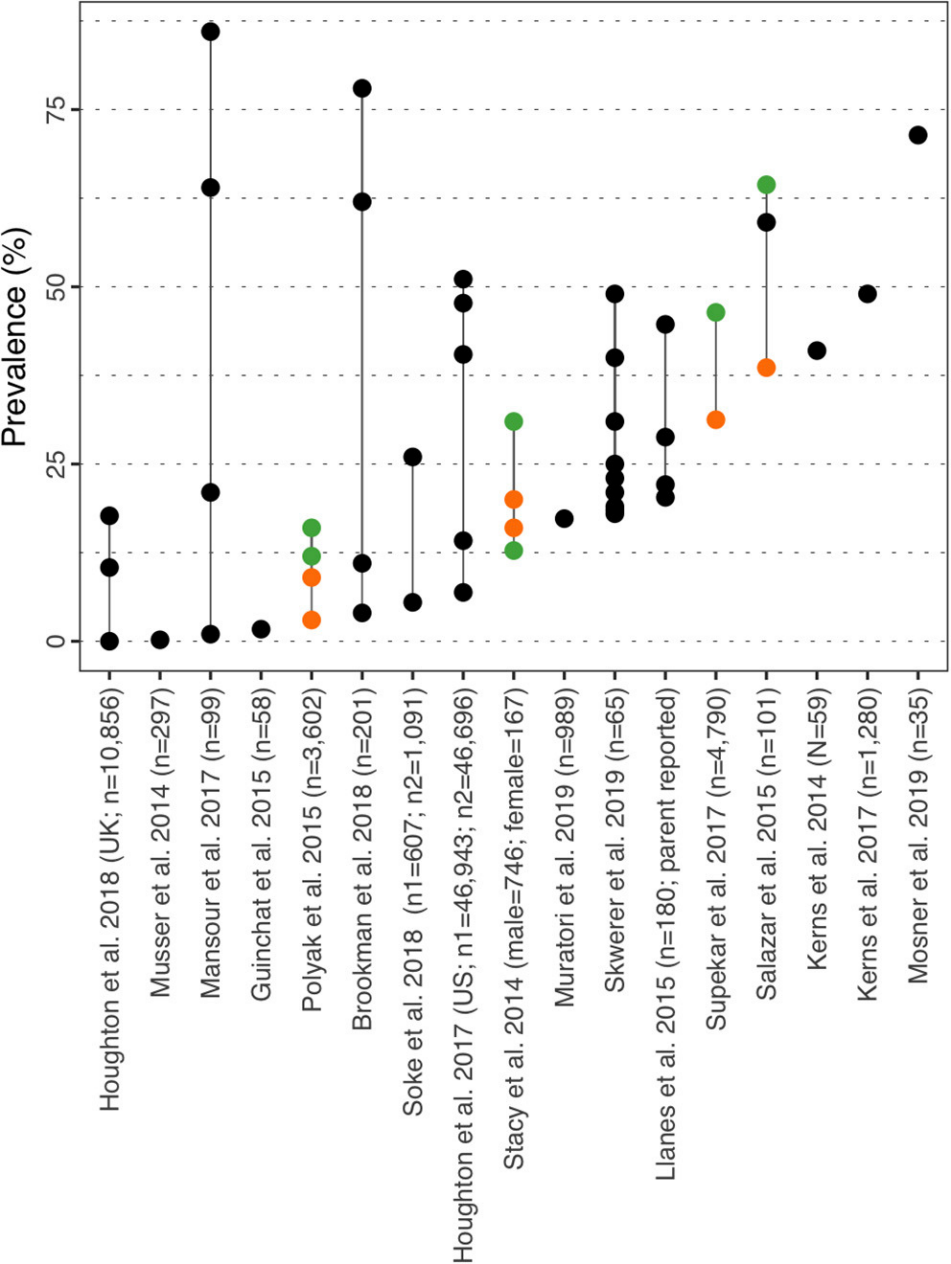
ADHD prevalence estimates. Each point reports a prevalence estimate for the related comorbidity. Studies with more than one data point indicate the estimate of the prevalence among different subgroup of the population. See Supplementary Table 4 for the description of the population used in each study. Sample sizes are shown in brackets above each study label. Orange mark: Female population only; Green mark: Male population only; Black mark: No sex distinction.

Prevalence for ADHD in ASD children ranged from 0.00% in children aged 3–4y (35) to 86.00% (Figure 3 and Supplementary Material 6) (36). Prevalence was further described by ADHD subtypes in Brookman-Frazee et al. (37), Mansour et al. (36), and Skwerer et al. (38) with a prevalence of 11.00, 21.00, and 40.00% respectively for the inattentive subtype, 4.00, 1.00, and 23.10% for the impulsive subtype and 62.00, 64.00, and 18.50% for both subtypes combined. In the US, Houghton et al. (39) studied the prevalence of co-morbidities in ASD using two insurance claims databases, one commercial database (Truven Health MarketScan® Commercial Database) and the Medicaid database. The authors reported the prevalence of ADHD for three age groups: 6.89/14.19% in 3–4y, 40.46/47.67% in 5–11y, and 47.73/51.08% in 12–17y (commercial/Medicaid). The authors conducted a similar study in the UK and leveraged the Clinical Practice Research Datalink database to estimate the prevalence of multiple co-morbidities while considering the same age groups. Prevalence for ADHD was 0.0% in children aged 3–4y, 10.40% in 5–11y, and 17.7% in the 12–17y group (35). In a UK study, Salazar et al. (40) reported the prevalence of ADHD to be 59.10% for the total population and by sex 64.40% (CI_95%_: 50.00–78.70%) in male children aged 4–8 y and 38.60% (CI_95%_: 23.90–53.40%) in female children. The authors also calculated a statistically significant odds ratio (OR) of 2.9 (CI_95%_: 1.2–6.9), confirming the disorder was more prevalent in male individuals. In a similar manner, Stacy et al. (32) also reported the prevalence of ADHD by sex, but also further stratified the disorder between “mild” and “moderate/severe” ADHD (32). Prevalence for mild ADHD in male/female children was 12.80/15.60% while prevalence of moderate/severe ADHD was 30.60/20.30%. Like in Salazar et al. the authors also calculated OR for each subtype, but none were statistically significant (OR mild ADHD = 1.03 [CI_95%_: 0.39–2.71]; OR moderate/severe ADHD = 0.56 [CI_95%_: 0.25–1.26]). Supekar et al. (41) assessed the influence of sex and age on the prevalence rates of comorbidities in ASD leveraging the STRIDE database and reported rates of co-morbid ADHD of 46.39 and 31.25% in males and females aged 0 to 18 years old respectively. Overall, ADHD appears more prevalent in male adolescents as sex was identified as a risk factor in Salazar et al. (40), and also confirmed in Brookman-Frazee et al. (37) who found that boys were 3.37 more likely to score positive for any ADHD disorder on the MINI-KID-P, while Soke et al. (42) found ADHD to be more prevalent in children aged 8y vs. 4y, with an adjusted OR = 4.78 (CI_95%_: 3.40–6.73).

#### Anxiety

**Figure 4:**
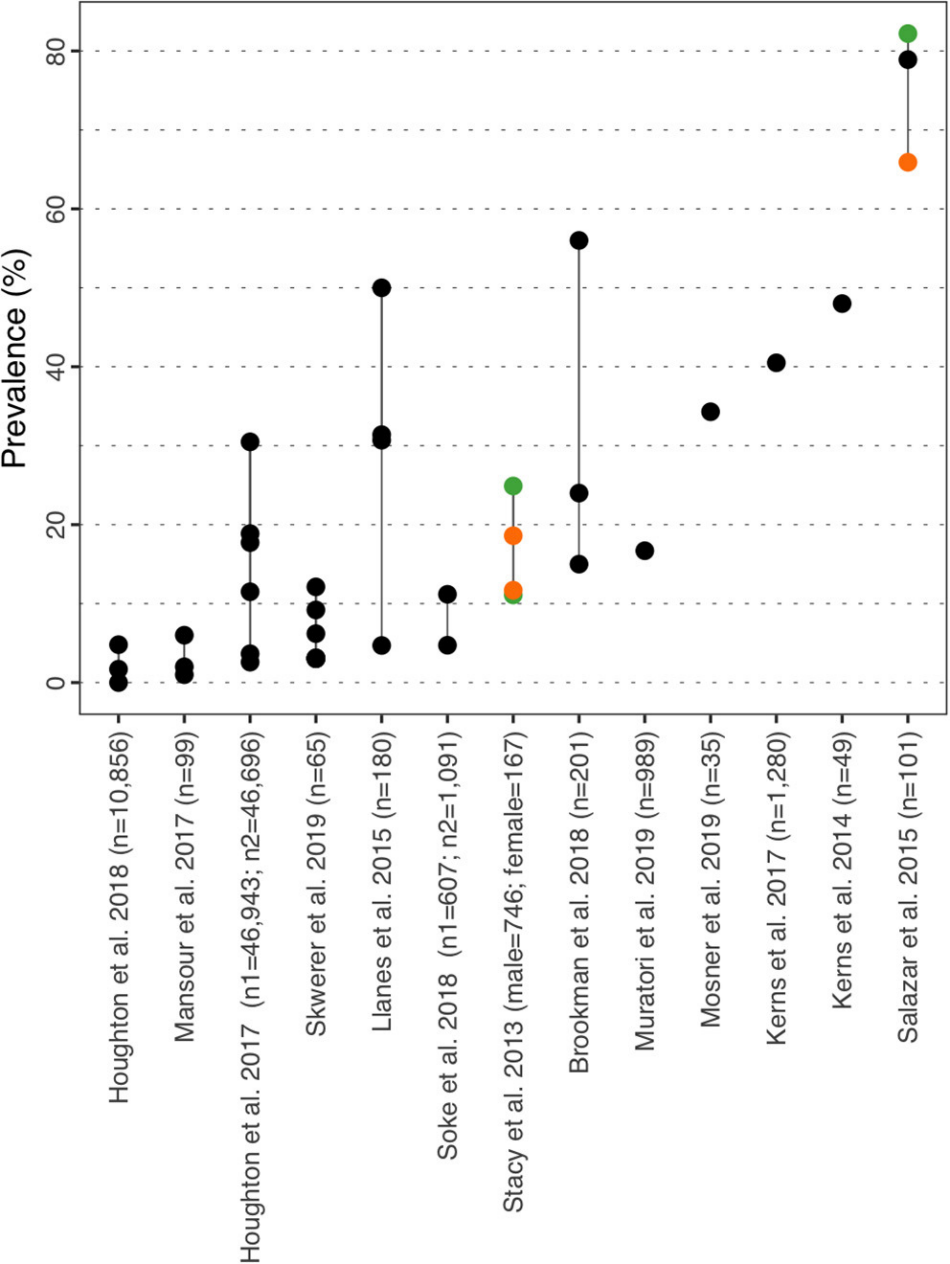
Anxiety prevalence estimates. Each point reports a prevalence estimate for the related comorbidity. Studies with more than one data point indicate the estimate of the prevalence among different subgroup of the population. See Supplementary Table 4 for the description of the population used in each study. Sample sizes are shown in brackets above each study label. Orange mark: Female population only; Green mark: Male population only; Black mark: No sex distinction.

Prevalence for anxiety varied between 0.00% in children aged 3–4y (35) and 82.20% in Salazar et al. (40) (Figure 4 and Supplementary Material 6). Similarly to ADHD, prevalence for anxiety was further described by subtypes in Brookman-Frazee et al. (37), Mansour et al. (36), and Skwerer et al. (38), although the subtypes considered were different in all three studies. However, all three authors reported prevalence for social phobia and separation anxiety disorder of 24.00, 6.00, 9.20, and 15.00, 1.00, 3.10% respectively. In the US, Houghton et al. (39) reported prevalence based on two cohorts (commercial/Medicaid) of 3.65/2.59% in 3–4y, 18.87/11.50% in 5–11y and 30.49/17.73% in 12–17y. The same study conducted in the UK showed prevalence of 0.00% in children aged 3–4y, 1.70% in 5–11y and 4.8% in the 12–17y group (35). In Salazar et al. (40), prevalence of any anxiety disorder was 65.90% in female vs. 82.20% in male, and the statistical analysis did not identify sex as a risk factor for this disorder [OR = 2.40 (CI_95%_: 0.90–6.00)]. However, an Intellectual Quotient (IQ) above 70 was identified as a risk factor for anxiety in ASD with a statistically significant OR of 2.90 (CI_95%_: 1.00–8.10). Salazar et al. (40) also provides prevalence for agoraphobia, specific phobia, panic disorder, generalized anxiety disorder and separation anxiety disorder all stratified by sex, IQ, age at assessment and ASD severity. Similar to ADHD, Stacy et al. (32) reported prevalence rates of comorbid mild and moderate/severe anxiety of 11.70 and 18.60% in females respectively and 11.10 and 24.90% in males respectively (no significant differences). Anxiety appears to be more prevalent in older children and age was identified as a risk factor in Soke et al. (42) [adjusted OR = 2.28 (CI_95%_: 1.57–3.39)]. In their paper, Salazar et al. (40) provide a rationale for the higher prevalence of anxiety disorders in older children with higher IQ and hypothesized that both factors expose children to more anxiety-provoking situations and engage in higher-cognitions.

#### Depressive Disorders

**Figure 5:**
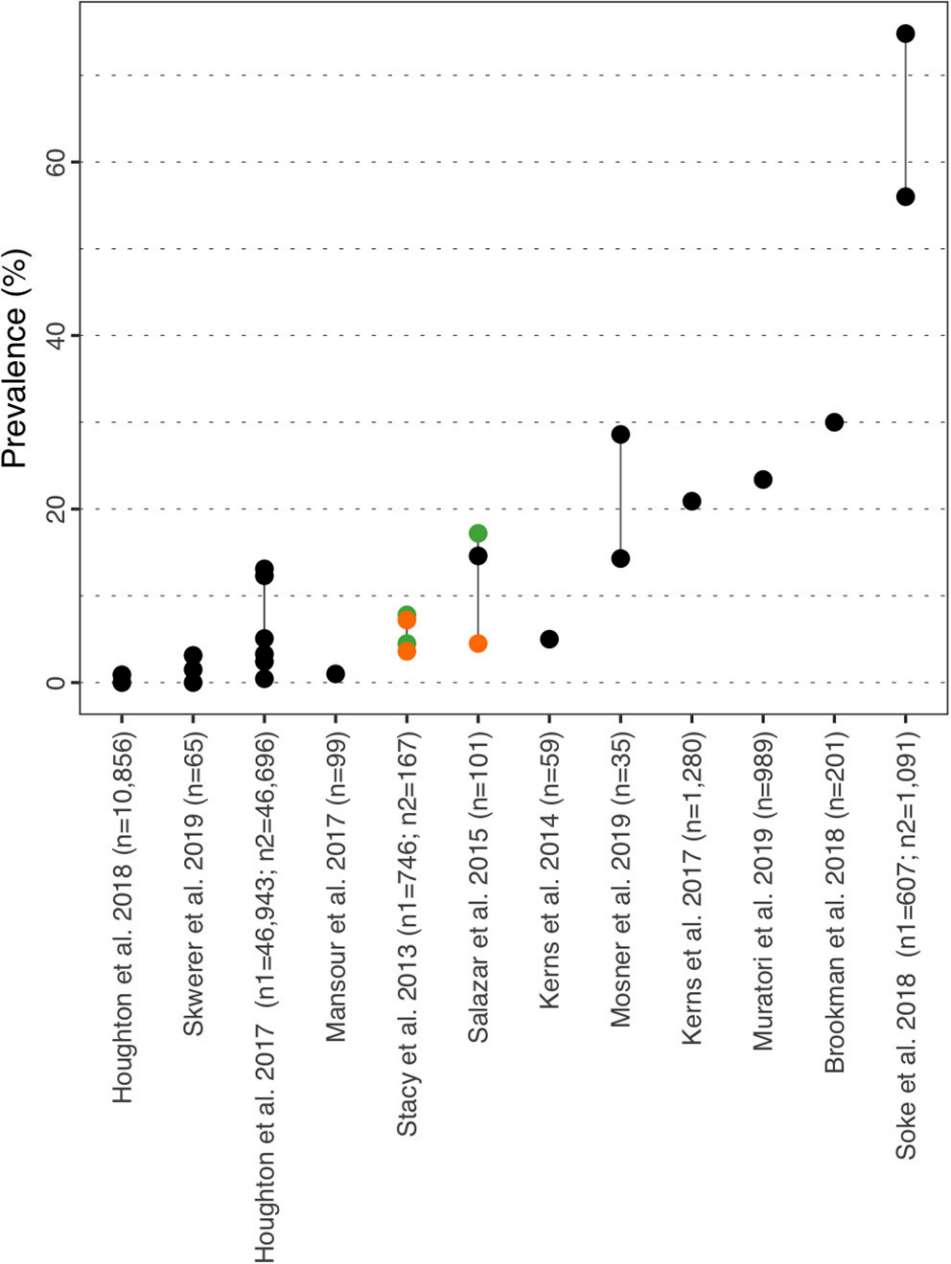
Depressive disorders prevalence estimates. Each point reports a prevalence estimate for the related comorbidity. Studies with more than one data point indicate the estimate of the prevalence among different subgroup of the population. See Supplementary Table 4 for the description of the population used in each study. Sample sizes are shown in brackets above each study label. Orange mark: Female population only; Green mark: Male population only; Black mark: No sex distinction.

Prevalence for depressive disorders varied from 0.00% in children aged 3–4y (35) to 74.80 % [any mood disorder] in ASD children aged 8y (42) (Figure 5 and Supplementary Material 6). Prevalence for depressive disorder subtypes (major depression disorder, dysthymia, maniac episode and hypomanic episode) are provided in Brookman-Frazee et al. (37), Mansour et al. (36), and Skwerer (38). In the US, Houghton et al. (39) reported prevalence of 0.44/3.28% in 3–4y, 2.42/5.08% in 5–11y and 13.11/12.31% in 12–17y (commercial/Medicaid). In the UK, Houghton et al. (35) estimated prevalence rates of 0.00% in children aged 3–4y and 0.90% in 5–17y. Similar to ADHD and anxiety, Soke et al. (42) identified age to be a risk factor for mood disorder with a prevalence of 74.80% in children aged 8y versus 56.00% in children aged 4y [adjusted OR = 1.25 (CI_95%_: 1.16–1.35)]. Salazar et al. (40) reported prevalence rates for major depression of 17.2% in males vs. 4.5% in females, but failed to confirm sex as a risk factor for this disorder (OR = 4.3 (CI_95%_: 0.8–22.4). Mild and moderate/severe depression was estimated at 7.2 and 3.6% respectively in females and 4.5 and 7.8% respectively in males in Stacy et al. (32) (difference not statistically significant). In Italy, Muratori et al. (43) reported a high prevalence rate for affective problems of 23.4% in children of mean age 44.01 months, whereas Soke et al. (42) reported a prevalence for mood disorders of 56.00% in children aged 4y. While Soke et al. (42) did not provide further insight on their results, Muratori et al. (43) mentioned their estimate was overestimated because of the tool used to assess the disorder (Child Behavior Checklist) that included items related to eating problem and sleep disorders to evaluate affective symptoms.

#### Epilepsy/Seizures

**Figure 6:**
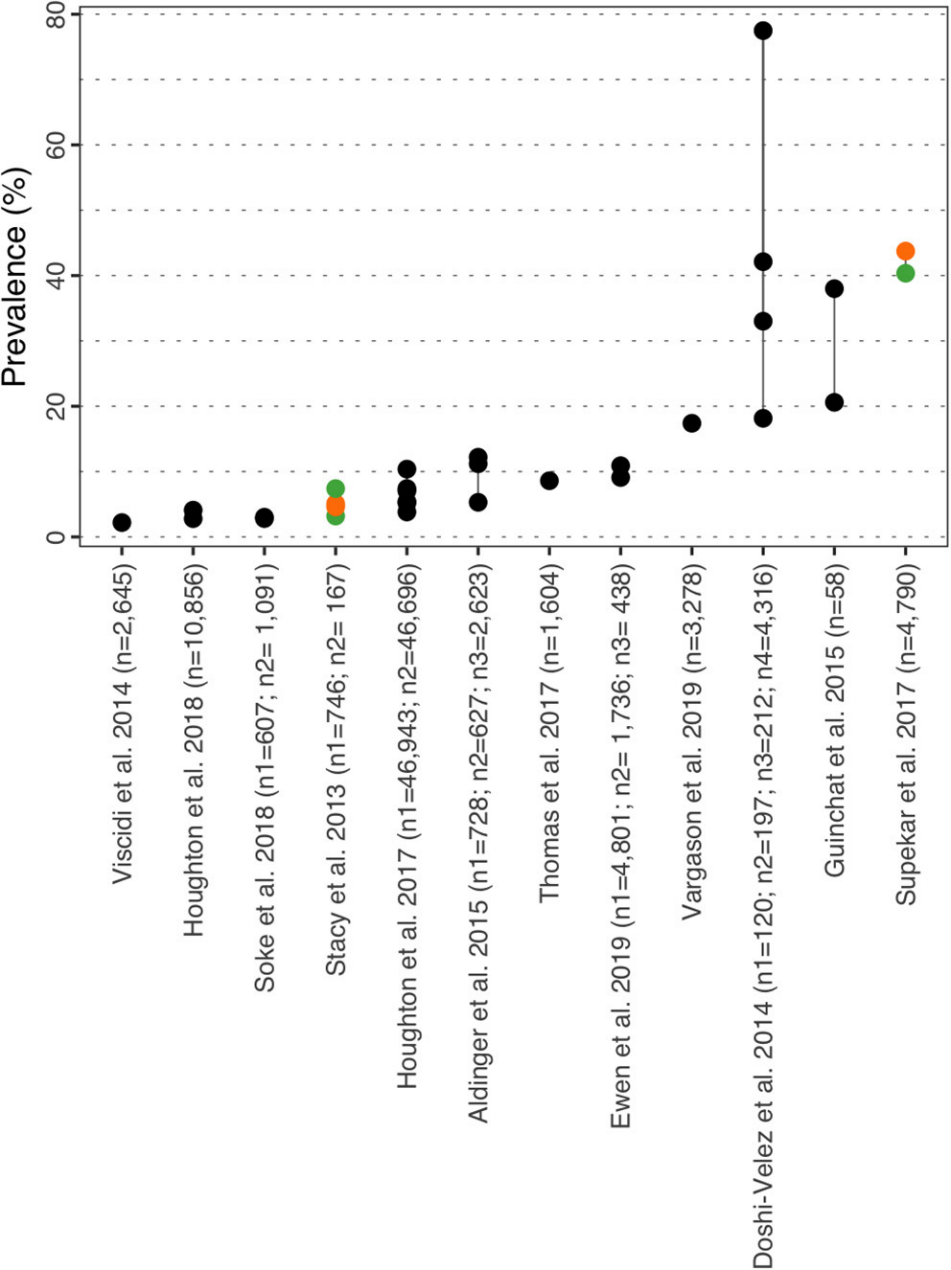
Epilepsy prevalence estimates. Each point reports a prevalence estimate for the related comorbidity. Studies with more than one data point indicate the estimate of the prevalence among different subgroup of the population. See Supplementary Table 4 for the description of the population used in each study. Sample sizes are shown in brackets above each study label. Orange mark: Female population only; Green mark: Male population only; Black mark: No sex distinction.

Prevalence for epilepsy/seizure in ASD children ranged from 2.80% in children aged 3–11y (35) to 77.50% in a subgroup of ASD individuals characterized by rate of ID in Doshi-Velez (44) (Figure 6 and Supplementary Material 6). In the US, Houghton et al. (39) reported estimates of 3.82/5.41% in 3–4y, 5.21/7.40% in 5–11y and 7.04/10.38% in 12–17y (commercial/Medicaid). In the UK prevalence was 2.80% in children aged 3–11y and 4.10% in 12–17y (35). Soke et al. (42) reported rates of 2.81% and 3.02% in children aged 4y and 8y respectively, but did not identify age as a risk factor (adjusted OR = 0.99 (CI_95%_: 0.54–1.84). Superkar et al. (41) reported rates of 40.36% and 43.75% in male and female with ASD aged 0–18y respectively and observed statistically higher rates in females with ASD, however this observation only applied to the whole study population aged 0 to 35y and more. Stacy et al. (32) reported prevalence rates of comorbid mild and moderate/severe epilepsy of 5.10 and 4.60% in females respectively and 7.40 and 3.20% in males respectively (without significant difference). The publication of Ewen et al. (45) specifically studied epilepsy in children with ASD and considered two cohorts from an online research registry (interactive autism network). In one cohort, the child with ASD questionnaire (CAQ) was used to estimate prevalence while the birth and ASD diagnosis history questionnaire (BDQ) was used in the second cohort. The authors reported prevalence of 9.10% in the CAQ cohort and 10.90% in the BDQ cohort and also demonstrated that ID, language deficit, ASD severity, and motor performance abnormalities were risk factors for epilepsy after adjusting for age and sex, with ID being the most impactful parameter [relative risk [RR]_(CAQ)_ = 2.22 (CI_95%_: 1.77–2.79); RR_(BDQ)_ = 2.18 (CI_95%_: 1.57–3.02)]. In the CAQ cohort, the authors also established that female sex was associated with 40% increased risk for epilepsy compared to males.

#### Gastro-Intestinal Syndromes

**Figure 7:**
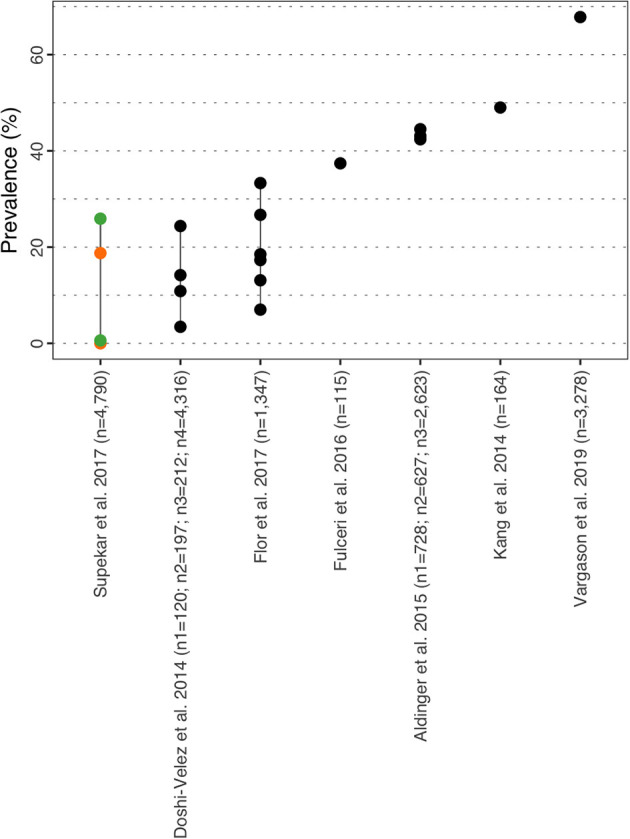
GI prevalence estimates. Each point reports a prevalence estimate for the related comorbidity. Studies with more than one data point indicate the estimate of the prevalence among different subgroup of the population. See Supplementary Table 4 for the description of the population used in each study. Sample sizes are shown in brackets above each study label. Orange mark: Female population only; Green mark: Male population only; Black mark: No sex distinction.

Prevalence of GI varied from 0.0% for inflammatory bowel disorders in female with ASD aged 0–18y (41) to 67.80% of children in Vargason et al. (46) (Figure 7 and Supplementary Material 6). Superkar et al. (41) also reported comorbid rates for bowel disorders of 25.90% and 18.75% in males and females respectively. In Italy, Fulceri et al. (47) estimated the prevalence of seven GI symptoms (“constipated”, “diarrhea”, “not eat”, “nausea”, “painful bowel movements”, “stomach-aches”, “vomiting”) in pre-schoolers with ASD compared to a group of typically developed children. Assessment of symptoms was done using the Child Behavior Checklist 1^1/2^-5 (Italian version), and authors reported an overall prevalence of 37.40% (29.6% in males, 7.8% in females) vs. 14.80% in typically developed children (*p* = 0.0001). “Constipated” and “not eat” were most prevalent subtypes observed in the ASD group with 15.70 and 27.00% respectively, and both rates were significantly higher compared to the typically developed group. Similarly, the research by Kang et al. (48) specifically aimed at assessing the frequency and characteristics of symptoms of GI disorders in 186 children with a median age of 7y (range 2–18y) estimated a prevalence rate of 49.00% for GI symptoms and also for the following subtypes: diarrhea (22%; *n* = 37), constipation (26%; *n* = 43), bloating (13%; *n* = 20) and gastro-enteric reflux/vomiting (10%; *n* = 16). The authors also demonstrated that the presence of any GI disorder was associated with significantly higher rates of sleep disorders (*p* = 0.001) and food intolerance (*p* < 0.001).

#### Hearing Impairment

**Figure 8:**
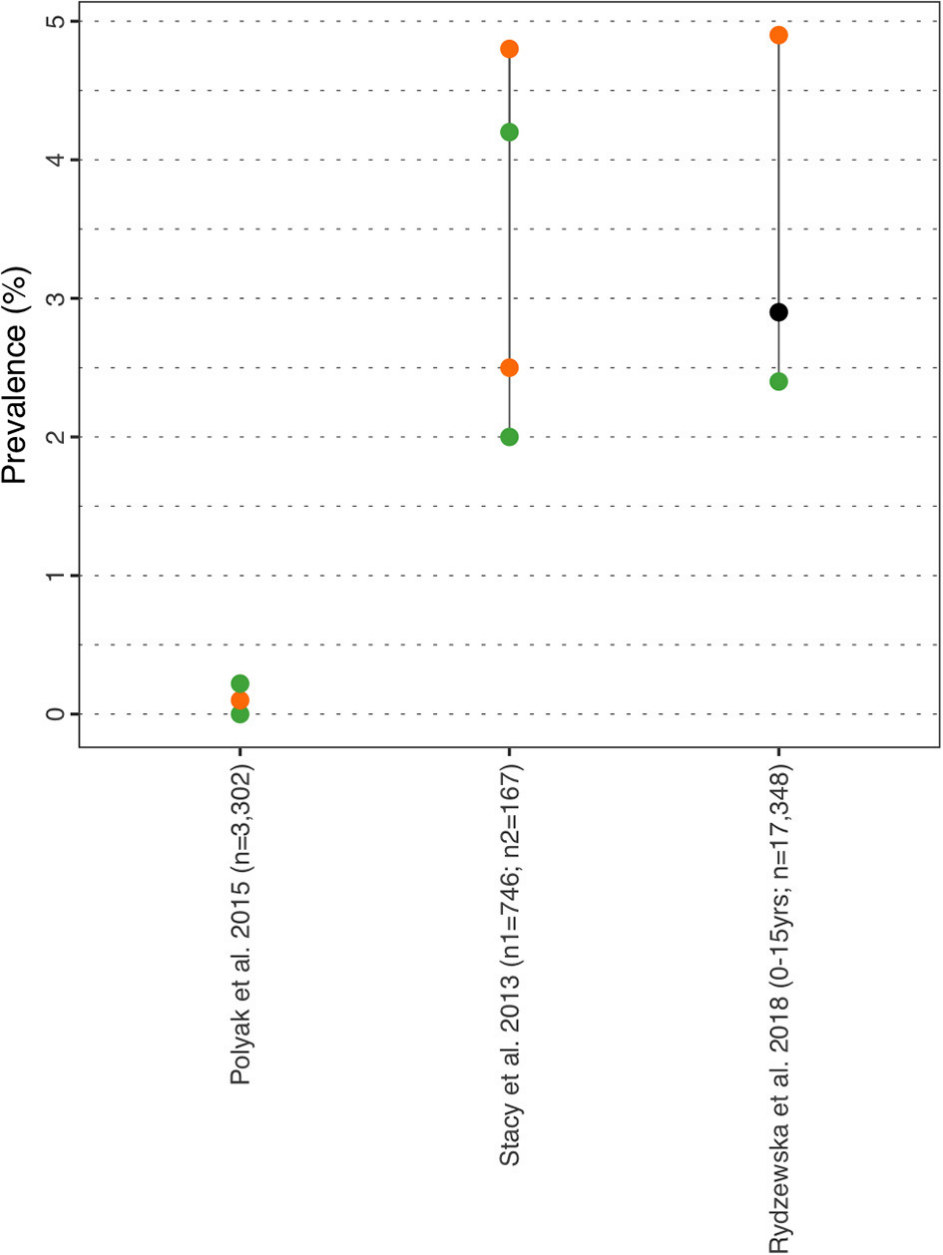
Hearing prevalence estimates. Each point reports a prevalence estimate for the related comorbidity. Studies with more than one data point indicate the estimate of the prevalence among different subgroup of the population. See Supplementary Table 4 for the description of the population used in each study. Sample sizes are shown in brackets above each study label. Orange mark: Female population only; Green mark: Male population only; Black mark: No sex distinction.

Prevalence of hearing impairment ranged from ~0% (females) in Polyak et al. (31) a study investigating sex bias in ASD comorbidities to 4.90% in Rydzewska et al. (34) (Figure 8 and Supplementary Material 6). The latter was a Scottish study investigating comorbid conditions in ASD vs. general population (0–24 years). Deafness or partial hearing loss was observed in 2.90% (2.40/4.90% in males/females) vs. 0.50% in the general population for the younger age group (0–15 years), and 3.90% (3.20/6.30% in males/females) vs. 0.80% for the older age group (16–24 years). The study concluded that hearing impairment was nine times more prevalent in the autistic individuals compared to the general population. Finally, Stacy et al. (32) reported prevalence rates of comorbid mild and moderate/severe hearing problem of 2.50 and 4.80% in females respectively and 2.00 and 4.20% in males respectively (without significant difference).

#### Intellectual Disability

**Figure 9:**
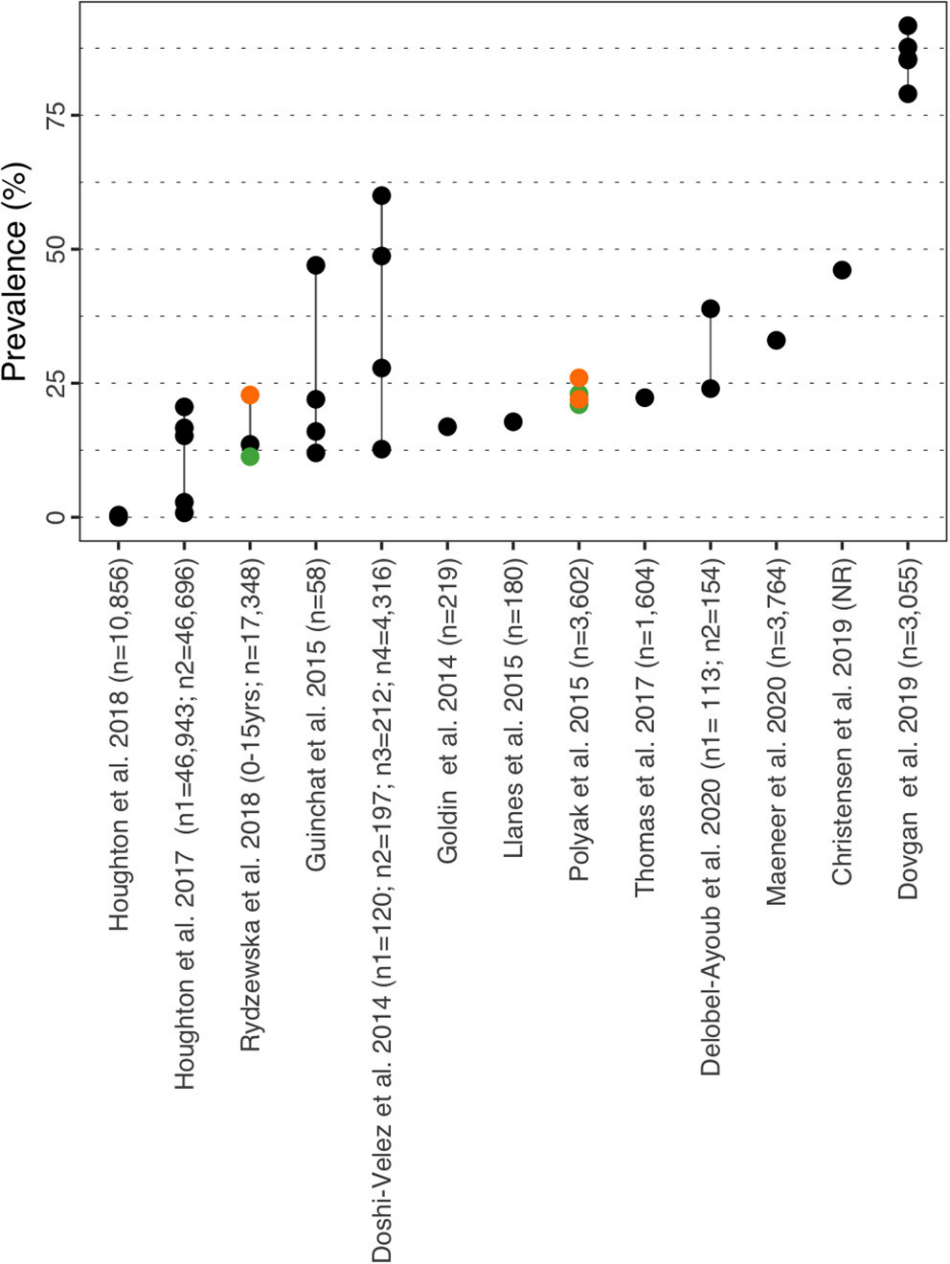
Intellectual disability prevalence estimates. Each point reports a prevalence estimate for the related comorbidity. Studies with more than one data point indicate the estimate of the prevalence among different subgroup of the population. See Supplementary Table 4 for the description of the population used in each study. Sample sizes are shown in brackets above each study label. Orange mark: Female population only; Green mark: Male population only; Black mark: No sex distinction.

ID was defined by an IQ score below 70 in all studies. Prevalence of ID in ASD children ranged from 0.00% in children aged 3–4y (35) to 91.70% in female ASD aged 0-18y (Figure 9 and Supplementary Material 6) (49). Prevalence of ID in Dovgan and Mazurek (49) was estimated based on telephone surveys aimed at parents of children with ASD and asked the following question: ‘have “a doctor or other health care provider ever told [him/her] that [the child] had” other specific co-occurring conditions?’. The study population was stratified by the number of co-morbidities, and the prevalence of 91.7% corresponded to the subpopulation of children who had ASD associated with three co-morbidities. In the US, Christensen et al. (15) and Maenner et al. (26) assessed the prevalence of ID in ASD children aged 4y and 8y respectively based on the ADDM network and reported rates of 46.10% and 33.00%. In Scotland, Rydzewska et al. (31) prevalence rates of 11.30% in male and 22.80% in female children aged 0-15y based on data from the Scotland's Census 2011. Additionally, the author reported that ASD was a strong predictor for ID, with an OR of 15.70 (CI_95%_: 13.40–18.50). Finally, Goldin (50) aimed at assessing the impact of ID on the presence of comorbid symptoms (tantrum behavior, repetitive behavior, worry/depressed, avoidant behavior, under-eating, conduct disorder, over-eating) in children with ASD and did not establish any significant impact.

#### Sleep Disorders

**Figure 10:**
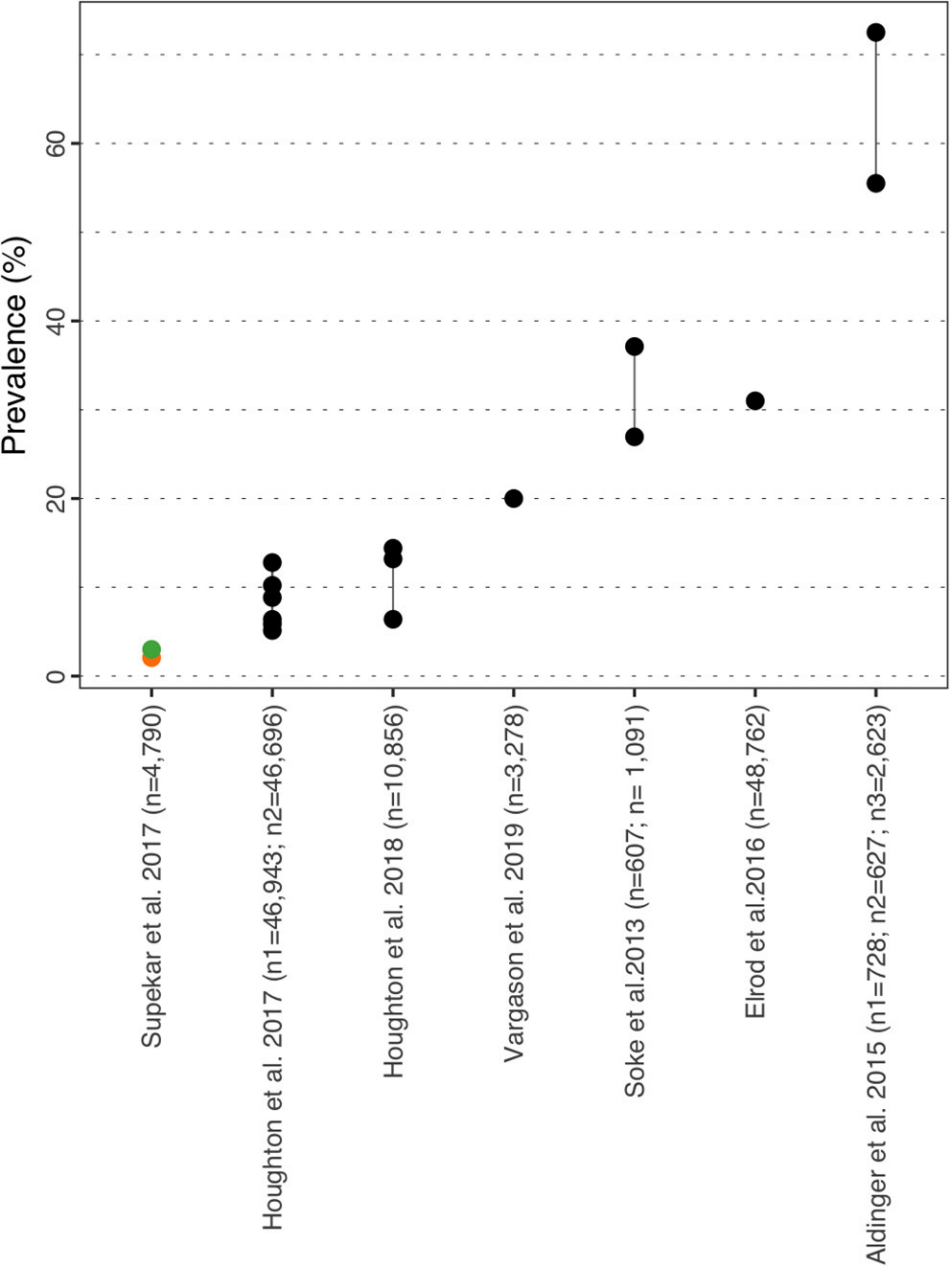
Sleep disorder prevalence estimate. Each point reports a prevalence estimate for the related comorbidity. Studies with more than one data point indicate the estimate of the prevalence among different subgroup of the population. See Supplementary Table 4 for the description of the population used in each study. Sample sizes are shown in brackets above each study label. Orange mark: Female population only; Green mark: Male population only; Black mark: No sex distinction.

Prevalence for sleep disorders varied from 2.08% in ASD females aged 0–18y (41) to 72.50% in ASD children with a mean age of 9y (Figure 10 and Supplementary Material 6) (51). In the US, Houghton et al. (39) reported prevalence of 6.39/12.79% in 3–4y, 5.87/10.22% in 5–11y and 5.14/8.85% in 12–17y (commercial/Medicaid). In the UK, Houghton et al. (35) estimated prevalence at 6.40% in children aged 3–4y, 13.20% in children aged 5–11y and 14.40% in 12–17y. Similar to previously mentioned co-morbidities, age was identified as a risk factor (adjusted OR = 1.34 [CI_95%_: 1.15–1.56]) for sleep abnormalities in Soke et al. (42) which estimated prevalence of 26.95% in children aged 4y and 37.12% in children aged 8y. In a retrospective case-cohort study based on the military health system database, Elrod et al. (52) analyzed a cohort of 48,762 children fulfilling the study case definition for ASD, and 31.00% displayed an ICD-9 code for one sleep disorder (vs. 14.00% in the control group). The most prevalent subtypes of sleep disorders were: sleep disorder–not otherwise specified (21.72%; *n* = 10,593), insomnia (9.52%) and sleep disorder breathing (9.26%). The authors calculated a RR of receiving a sleep disorder diagnosis of 1.97 in children with ASD (CI_95%_: 1.91–2.02). Aldinger et al. (51) reported the above mentioned rate of 72.50% for sleep disorder which correspond to the rate calculated for the Simons Simplex Collection cohort [family-based cohort consisting of families having one child with ASD (simplex)]. However the study neither reported the method used to estimate sleep disorders nor commented on this prevalence rate. Finally, Superkar et al. (41) reported rates of 3.01 and 2.08% in male and female with ASD aged 0–18y respectively.

#### Vision Impairment

**Figure 11:**
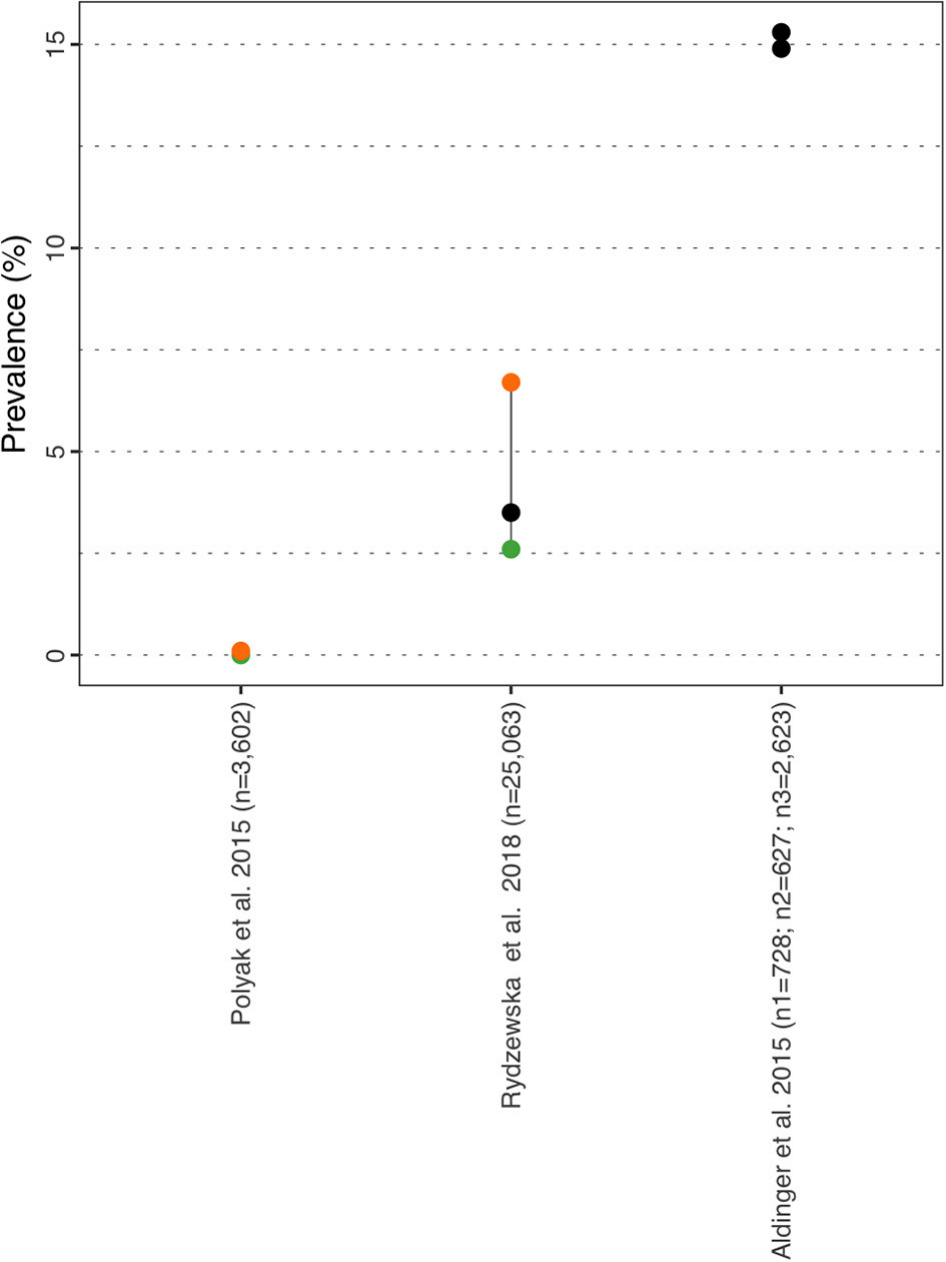
Vision impairment prevalence estimate. Each point reports a prevalence estimate for the related comorbidity. Studies with more than one data point indicate the estimate of the prevalence among different subgroup of the population. See Supplementary Table 4 for the description of the population used in each study. Sample sizes are shown in brackets above each study label. Orange mark: Female population only; Green mark: Male population only; Black mark: No sex distinction.

Prevalence for vision impairment ranged from ~0% (males and females) in Polyak et al. (34) study examining the impact of sex bias on comorbidities in ASD, to 14.9% in Aldinger et al. (51) (Figure 11 and Supplementary Material 6). Aldinger et al. (51) compared a family based cohort that predominantly consists of families with more than one child with ASD (multiplex) vs. a family with one ASD child (simplex). Vision problems were observed in the multiplex cohort (14.9 and 15.3% complete sample). Rydzewska et al. (31) reported estimates of 3.50% (*n* = 599) in children aged between 0 to 15 years old (males = 2.6%; females = 6.7%) for the outcome “blindness/partial sight loss”, and 3.80% in the 16 to 24 year old group (males = 3.0%; females = 6.4%).

## Discussion

The objective of this study was to describe the clinical burden associated with ASD in children and adolescents based on recent prevalence data and trends over time, as well as understanding the prevalence of nine co-morbidities associated with ASD and potential differences by age and gender. Our research focused on children and adolescents with ASD (age <18y) and considered studies conducted in the US, France, Germany, Italy, Spain, and UK.

Our first review found the latest prevalence data for each country, with 1.70 and 1.85% in the US in children aged 4y and 8y respectively, while prevalence of ASD in Europe ranged from 0.38% in Germany (0–24 years) to 1.55% (3–5 y) in Spain. Of note, Fuentes et al. (53) recently published the results of the ASDEU initiative for Spain and reported a prevalence of 0.59% in children aged 7–9y, which is substantially lower than the one reported by Morales-Hidalgo et al. (18). Although the overall screening and diagnosis processes are similar in both studies, the difference can be explained by the difference in sample sizes [10,512 participants in Fuentes et al. (53) vs. 2,827 in Morales-Hidalgo et al. (18)]. Prevalence data for the US is based on the ADDM network, an active and national surveillance system that reported the prevalence of ASD since 2000. However, the methodologies used to estimate the prevalence of ASD in the five European countries were all different, whether in terms of age group considered or method to estimate prevalence. The absence of a global European surveillance network implies that each country individually assesses the prevalence in its own population. As such estimates between European countries are highly heterogeneous, which makes interpretation and comparison of these differences challenging. These differences in prevalence can also be extended to country healthcare systems as most countries in Europe have their own way of diagnosing, apprehending and managing ASD. Our findings are in accordance with those of Chiarotti and Venerosi (5) who conducted a narrative review on the worldwide prevalence of ASD, and identified the same studies. Similarly, Autism Europe estimates between 1.0 and 1.5% the prevalence of ASD in Europe, which is in line with the prevalence of 1.22% reported by ASDEU (5). The higher prevalence reported in ASDEU compared to the five above mentioned European countries can be explained by the inclusion of countries with high prevalence such as Iceland who reported a prevalence of 2.68%, possibly due to the limited population of the country. Male to female ratios in our review varied between 2.7 in Germany and 6.5 in the UK and is consistent with previous studies that are supportive of a 4:1 ratio. Studies have shown that prevalence is potentially underestimated in females, as autistic females could better escape diagnosis or ASD may only become evident in adolescence when social demands increase (12, 54). Overall, current evidence is supportive of a global increase in ASD over the past years, while suggesting that the augmentation is not specific to a particular region or healthcare system, but is observed at a broader level. Possible hypothesis behind this progression have been well formulated in Myers et al. (21) who suggested that the increase in prevalence is a result of better professional and public awareness of ASD rather than a true augmentation in the number of cases. Another explanation could be the focus of clinical practice on subtle expression of symptoms in high-functioning autistic children over the past years. Finally, internal and external causes such as parental age or exposure to drugs like thalidomide or valproic acid during pregnancy could also be associated to this augmentation (55). However, the current evidence around the role of some of these factors is still unclear and represents active areas of research (4).

It is possible that prevalence estimates in ASD are underestimated for multiple reasons, the first one lying in complexity of the disorder itself. ASD is a complex disorder associated with a wide array of phenotypes and numerous levels of severity. International guidelines on ASD suggest that each case should be considered individually and diagnosis made by a multidisciplinary team. However, availability of multidisciplinary teams and expert clinicians is rare, leading to extended waiting list and children escaping diagnosis. Underestimation of the prevalence of ASD could also be attributed to the multiplicity of diagnostic tools available. Such tools are commonly used to support diagnosis and complete clinical assessment, but no gold standard officially exists. Although, the combination of the ADOS and ADI-R is commonly reported, both tools require time and skills to administer which can lead to inaccurate evaluations. On the other hand, it is also possible for prevalence to be overestimated in case of misdiagnosis with other disorders and in situations where ASD is associated with services and care that would not be otherwise granted for other disorders. Evidence as to whether the switch from the DSM-IV to the DSM-5 algorithm of ASD has impacted on its prevalence is conflicting (56, 57).

As for the prevalence of co-morbidities in ASD, our SLR included a total of 33 studies and highlighted wide prevalence ranges for each co-morbidity: 0.0–86% for ADHD (17/33 studies), 0.0–82.2% for anxiety (13/33 studies), 0.0–38.6% for depressive disorders (12/33 studies), 2.8–43.75% for epilepsy/seizures (12/33 studies), 0.0–49.0% for GI syndromes (7/33 studies), 0.0–87.8% for hearing impairment (3/33 studies), 0.0–91.7% for ID, 6.4–72.5% for sleep disorders (5/33 studies) and 0.0–15.3% for visual impairment (3/33 studies). Lower estimates of 0.0% for ADHD, anxiety, depressive disorders and ID originate from Houghton et al. (36) and correspond to prevalence estimates for the 3–4 year old age group. Similarly, estimates of 0.0% for both hearing and visual impairments are based on the results of Polyak et al. (34) although they correspond to a highly specific population of ASD children with rare copy-number variants. In their umbrella review, Hossain et al. reported ranges of 25.7–65.0% for ADHD, 1.5–54.0% for anxiety and 2.5–47.1% for depressive disorders while Lai et al. calculated a pooled prevalence of 28% for ADHD, 20% for anxiety, 11% for depressive disorders and 13% for sleep-wake disorders (6). However both studies considered pediatric and adult populations and different definitions for co-morbidities.

The literature suggests that the prevalence of some co-morbidities can be different with age and gender. Prevalence of ADHD and anxiety appear to increase until adolescence, with a decline observed when reaching adulthood (41, 42, 58). In addition, evidence is supportive of a higher prevalence of ADHD in male compared to female individuals (32, 37, 40). Epilepsy and depressive disorders were predominant in female individuals and slightly increasing with age (33, 38, 41, 42). Sleep disorders were more frequently observed in children than adolescents (41, 42). Evidence related to visual and hearing impairment is scarce and insufficient to formulate hypothesis about the impact of age. ADHD was found to be associated with a greater risk of having other psychiatric comorbidities in addition to lower cognitive functioning, more severe social impairment and greater delays in adaptive functioning (36). Comorbid epilepsy often occurs in conjunction with ID and was associated with worse behavioral and social outcomes, increased motor difficulties and more challenging behavior compared with ASD alone (59). Our review also gathered evidence that co-morbidities are more frequently observed in ASD individuals compared to typically developed population, which is in accordance with the current literature (7).

Similar to our findings for the prevalence of ASD, several factors should be considered when interpreting the heterogeneity in co-morbidity prevalence. First, the definition for a given co-morbidity differed across studies. This was observed particularly for anxiety disorders where claim database studies such as Houghton et al. (39) considered a wide array of disorders from anxiety states to dissociative disorders, whereas the prevalence reported in Stacy et al. (32) corresponds to “anxiety” without further information. As such, the presence of subtypes within a single co-morbidity is a factor for heterogeneity. Second, study design and methodology should be considered when interpreting our results as our review included a mix of cohort, cross-sectional and case-control studies. Although informative, prevalence rates from surveys should be interpreted carefully as they are subject to multiple bias from parents who often must confirm or infer the presence of a co-morbidity based on a single question. Similarly, claims database analyses do not provide information regarding the diagnostic process undertaken and instrument(s) used to confirm the presence of the co-morbidity. Finally, diagnostic ambiguity should be considered when interpreting prevalence of co-morbidities as it is often challenging to distinguish a co-morbidity from the presentation of autism. As an example, deficit in communication/interaction in autism can be interpreted as an anxiety disorder while conversely it can prevent from reporting symptoms potentially attributable to a co-morbidity.

Our study presents several limitations. First, a broad search term for comorbidity was considered and not terms for specific comorbidities. This approach allowed us to identify the most frequently observed comorbidities in ASD whose relevance was further confirmed by clinical experts. The second limitation lies in the decision to include studies reporting prevalence as a secondary outcomes. While these studies did provide relevant information, the rates extracted from these studies might include a selection bias by selecting specific subpopulations of children with ASD. Third, although the purpose of this study was to provide a descriptive review of the prevalence of ASD and its co-morbidities, additional research could involve analysis focusing on a single co-morbidity to estimate an overall prevalence rate while accounting for the heterogeneity across studies highlighted in our research. Fourth, our review focused on the most common co-morbidities reported in the literature but other co-morbidities can be observed in association with ASD such as dementia or allergic/autoimmune diseases. Finally, as our search strategy focused on children and adolescents in the US and five European countries, this study did not capture the evidence generated in other geographic regions as well as in adults with ASD.

Our research provides a descriptive review of the prevalence of ASD and its co-morbidities which can be valuable for clinicians as well as parents/guardians of children with ASD. Despite substantial heterogeneity in estimates, our results indicate that co-morbidities are highly prevalent in ASD, and should be kept under consideration when diagnosing and managing individuals with ASD. In addition, co-morbidities should be re-assessed regularly as evidence suggest that prevalence evolves over time, especially during transition ages such as entry to school or adolescence. Also, co-morbidities bring additional heterogeneity to the presentation of ASD, further advocating for personalized and tailored approaches to treatment and support. Finally, co-morbidities in ASD negatively impact the quality of life and life expectancy of individuals as well as representing a substantial economic burden. Results from Hirvikoski et al. (60) reported a 2.56-fold increased odds of mortality in individuals with ASD compared with matched general population while Hedgecock et al. (61) reported a negative correlation between quality of life and behavioral disorders in autistic children. Buescher et al. (9) estimated the mean annual cost of ASD to be $63,292 and $52,205 in children aged 0–5y and 6–17y respectively. However costs increased to $107,863 and $85,690 in children with ASD and co-morbid ID. Similarly, Houghton et al. (39) showed that psychiatric co-morbidities in ASD were associated with increased medication and increased GP interactions, thus leading to higher healthcare resource consumption and potentially higher rates of adverse events from medication.

Our findings have clear implications for the diagnostic process of young people referred for suspicion of ASD and for service organization. The relative high probabilities of psychiatric and somatic co-occurring conditions should be incorporated into clinical guidelines for assessing ASD. The assessment should not only be focused on identifying the core symptoms of ASD but also on the wider range of other psychiatric and somatic problems. To this end, systematic information from parents and teachers should be collected through validated broad band questionnaires that tap internalizing and externalizing behavior problems, such as the ASEBA Questionnaires (62), the Conners Questionnaires (63), or the Strengths and Difficulties Questionnaire (SDQ) (64). The diagnostic work-up should also include an interview about somatic signs and symptoms, and a standard physical exam. Preferably, clinical care should be organized around the patient and provided by a multidisciplinary team of health care professionals with expertise in complementary areas.

These findings are supportive of a global increase in ASD prevalence independent from regions and healthcare systems and call for stronger awareness within populations and healthcare policies. Our review also provides prevalence estimates for nine co-morbidities frequently associated with ASD and highlighted the importance of age and gender in prevalence of both ASD and its co-morbidities. Across Europe, there is still a need for studies applying a similar methodology when estimating prevalence in order to allow comparison across countries. This review highlights the substantial clinical burden associated with ASD, with co-morbidities further complicating evaluation, management and prognosis of ASD. Finally reliable estimates for prevalence of ASD and associated co-morbidities would support economic analysis and further assessment of burden in ASD.

## Data Availability Statement

The original contributions presented in the study are included in the article/Supplementary Material, further inquiries can be directed to the corresponding author/s.

## Author Contributions

All authors were involved in the conception and design of the study. Data collection and analysis was conducted by CB and RC. Writing of the manuscript was done by CB. All authors reviewed and approved the manuscript.

## Funding

This study was funded by Servier.

## Conflict of Interest

CB and RC are employees of Syneos Health. Syneos Health was contracted by Institut de Recherche Servier to conduct this study. JB received honoraria from Institut de Recherche Servier for clinical expertise. FP-B and RS are employees of Servier.

## Publisher's Note

All claims expressed in this article are solely those of the authors and do not necessarily represent those of their affiliated organizations, or those of the publisher, the editors and the reviewers. Any product that may be evaluated in this article, or claim that may be made by its manufacturer, is not guaranteed or endorsed by the publisher.

## Abbreviations

ADDM: Autism and Developmental Disabilities Monitoring
ADI-R: Autism Diagnostic Interview-Revised
ADOS: Autism Diagnostic Observation Schedule
ASD: Autism Spectrum Disorder
ASDEU: Autism Spectrum Disorder in Europe
BDQ: Birth and ASD Diagnosis history Questionnaire
CAQ: Child with ASD Questionnaire
CI: Confidence Interval
DSM: Diagnostic and Statistical Manual of Mental Disorders
GI: Gastro Intestinal syndrome
ICD: International Classification of Diseases
ID: Intellectual Deficit
IQ: Intellectual Quotient
M/F: Male/Female ratio
OR: Odd Ratio
PICOS, Population, Intervention, Comparator, Outcome: Study
RR: Relative Risk
SCQ: Social Communication Questionnaire
SLR: Systematic Literature Review
TNF: Teacher Nomination Form
UK: United Kingdom
US: United States.
