# Supplementary material for: Prevalence of Autism Spectrum Disorder and Co-morbidities in Children and Adolescents: A Systematic Literature Review
Source: Front Psychiatry. 2021 Oct 27;12:744709. doi: 10.3389/fpsyt.2021.744709 (PMC8579007; doi:10.3389/fpsyt.2021.744709)
Supplement: Supplementary file 1 [file Table_1.docx]

Supplementary Material

[Supplement 1: Search strategy (Prevalence of ASD) 2](#_Toc81411239)

[Supplement 2: Search strategy (Prevalence of co-morbidities) 3](#_Toc81411240)

[Supplement 3: PRISMA diagram prevalence of ASD 4](#_Toc81411241)

[Supplement 4: Prevalence of co-morbidities in ASD – Characteristics of studies 5](#_Toc81411242)

[Supplement 5: Prevalence of co-morbidities in ASD – Summary of results 11](#_Toc81411243)

[Supplement 6: Prevalence of co-morbidities in ASD – Main results 15](#_Toc81411244)

Supplement 1: Search strategy (Prevalence of ASD)

|  | PubMed |  |  | Embase |
| --- | --- | --- | --- | --- |
| # | **Search terms** |  | **#** | **Search terms** |
| 1 | "Child Development Disorders, Pervasive"[Mesh] |  | **1** | 'autism'/de |
| 2 | "asperger syndrome"[Title/Abstract] |  | **2** | 'asperger syndrome'/de |
| 3 | "autism spectrum disorder"[Title/Abstract] |  | **3** | 'autism spectrum disorder':ab,ti |
| 4 | "autis*"[Title/Abstract] |  | **4** | autis*:ti,ab |
| 5 | "ASD"[Title/Abstract] |  | **5** | ASD':ti,ab |
| 6 | "childhood disintegrative disorder"[Title/Abstract] |  | **6** | 'childhood disintegrative disorder' |
| 7 | "PDD"[Title/Abstract] |  | **7** | pdd:ti,ab |
| 8 | "pervasive developmental disorder not otherwise specified"[Title/Abatract] |  | **8** | pervasive developmental disorder not otherwise specified'/de OR 'pdd nos'/de |
| 9 | "pdd-nos"[Title/Abatract] |  | **9** | pervasive developmental disorder not otherwise specified'/de OR 'pdd nos'/de |
| 10 | "autism"[Title/Abstract] |  | **10** | autism:ti,ab |
| 11 | or/1-10 |  | **11** | or/1-10 |
| 12 | "Epidemiology"[Mesh] |  | **12** | epidemiology'/de |
| 13 | Epidemiology[Title/Abstract] |  | **13** | incidence'/mj |
| 14 | "Incidence"[Mesh] OR "Incidence"[Title/Abstract] |  | **14** | 'prevalence'/mj |
| 15 | "Prevalence"[Mesh] OR "Prevalence"[Title/Abstract] |  | **15** | diagnosis'/mj |
| 16 | "Diagnosis"[Title] |  | **16** | tool*:ti |
| 17 | "Tool*"[Title] |  | **17** | scale*:ti |
| 18 | "Scale*"[Title] |  | **18** | OR/12-17 |
| 19 | or/12-18 |  | **19** | 11 AND 18 |
| 20 | 11 AND 19 |  | **20** | Limit to humans |
| 21 | Limit to humans |  | **21** | Limit to last 10 years |
| 22 | Limit to last 10 years |  | **22** |  |

Supplement 2: Search strategy (Prevalence of co-morbidities)

|  | PubMed |  |  | Embase |
| --- | --- | --- | --- | --- |
| # | **Search terms** |  | **#** | **Search terms** |
| 1 | "Child Development Disorders, Pervasive"[Mesh] |  | **1** | 'autism'/de |
| 2 | "asperger syndrome"[Title/Abstract] |  | **2** | 'asperger syndrome'/de |
| 3 | "autism spectrum disorder"[Title/Abstract] |  | **3** | 'autism spectrum disorder':ab,ti |
| 4 | "autis*"[Title/Abstract] |  | **4** | autis*:ti,ab |
| 5 | "ASD"[Title/Abstract] |  | **5** | ASD':ti,ab |
| 6 | "childhood disintegrative disorder"[Title/Abstract] |  | **6** | 'childhood disintegrative disorder' |
| 7 | "PDD"[Title/Abstract] |  | **7** | pdd:ti,ab |
| 8 | "pervasive developmental disorder not otherwise specified"[Title/Abstract] |  | **8** | pervasive developmental disorder not otherwise specified'/de OR 'pdd nos'/de |
| 9 | "pdd-nos"[Title/Abatract] |  | **9** | autism:ti,ab |
| 10 | "autism"[Title/Abstract] |  | **10** | or/1-9 |
| 11 | or/1-10 |  | **11** | risk factor'/mj |
| 12 | "Risk Factors"[Title/Abstract] |  | **12** | clinical burden' |
| 13 | "Comorbidity"[Mesh] |  | **13** | comorbidity'/de |
| 14 | "clinical burden"[Title/Abstract] |  | **14** | mortality'/de |
| 15 | "Mortality"[Mesh] |  | **15** | attitude'/mj OR 'attitude*':ti,ab |
| 16 | Mortality[Title/Abstract] |  | **16** | institutionalization'/mj OR 'institutionali*ation':ti,ab |
| 17 | "Attitude*"[Title/Abstract] |  | **17** | or/11-16 |
| 18 | "Institutionalization"[Mesh] OR Institutionalisation[Title/Abstract] |  | **18** | 10 AND 17 |
| 19 | or/12-19 |  | **19** | Limit to humans |
| 20 | 11 AND 20 |  | **20** | Limit to last 10 years |
| 21 | Limit to humans |  | **21** |  |
| 22 | Limit to 10 Years |  | **22** |  |

Supplement 3: PRISMA diagram prevalence of ASD


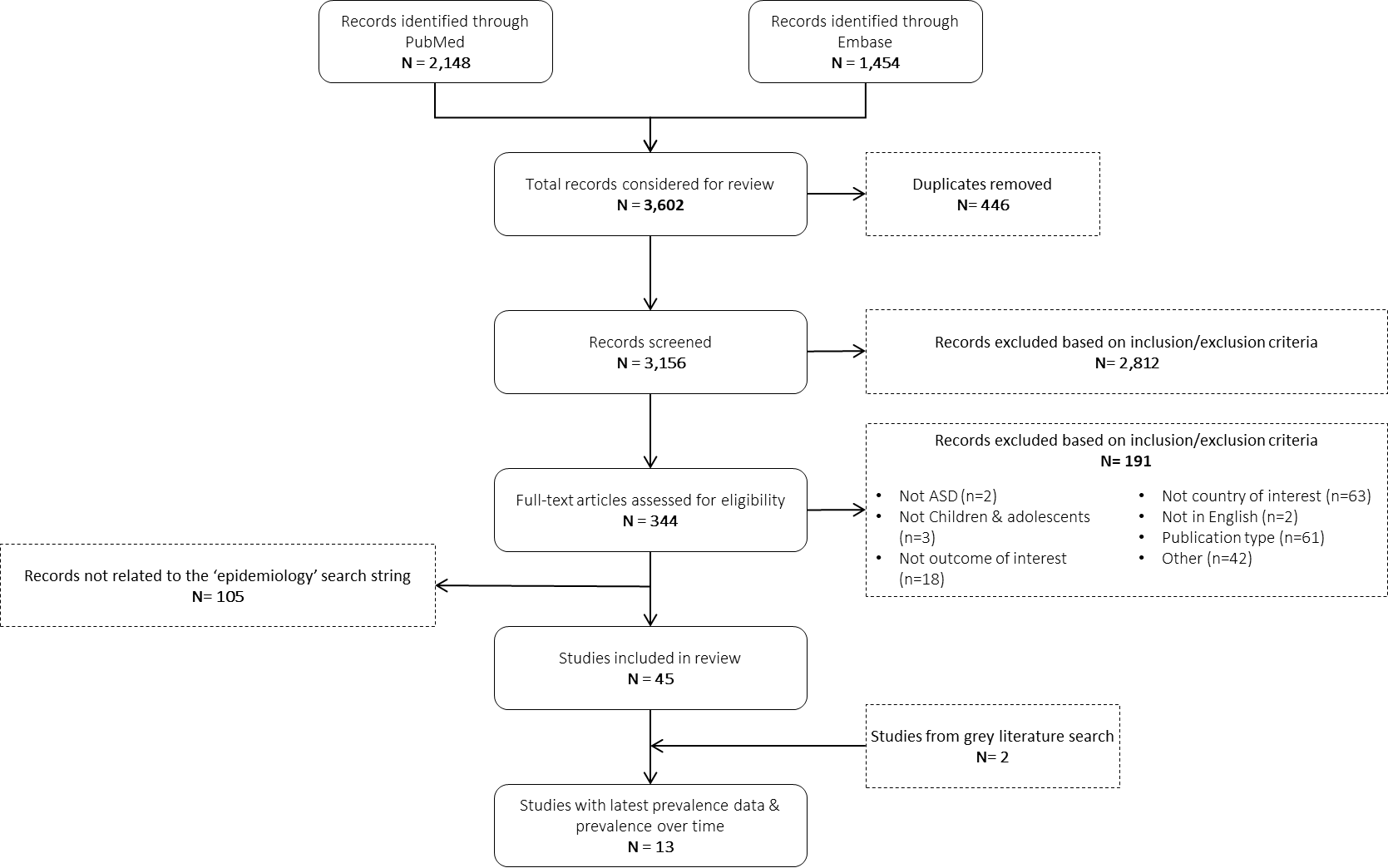


Supplement 4: Prevalence of co-morbidities in ASD – Characteristics of studies

| Author (Year) | Prevalence as primary outcome | Age group(s) | % male | Data source | ASD population |
| --- | --- | --- | --- | --- | --- |
| Aldinger *et al.* (2015) US | No | AGRE cohort (multiplex): M= 9.2y (SD= 5.1)  AGRE cohort (cluster analysis sample): M= 9.3y (SD= 5.2)  SSC cohort: M= 9.0y (SD= 3.6) | AGRE cohort (multiplex): 78.3%  AGRE cohort (cluster analysis sample): 79.7%  SSC cohort: 86.7% | AGRE  SSC | - Pedigree file - Medical history file - ADI-R - SRS - VABS-II - Stanford-Binet Intelligence Scale |
| Brookman-Frazee *et al.* (2018) US | Yes | M= 9.13y (SD=2.44)  Range: 4-14y | 84% | Population from a community effectiveness trial | - Cognitive Standard Score= 88.47 (16.53) - ADOS-2 Comparison Score= 7.06 (2.02) - SRS-2   - Total T-Score= 79.98 (11.15)   - T-Score ≥ 60= 95% (n=190) - ECBI Intensity T-Score= 63.01 (10.44)   - T-Score ≥ 60= 63% (n=125) |
| Christensen *et al.* (2019) US | Yes | 4y | 46.7% | ADDM network (2014) | Case determination is a two-phase process:   1. Review and abstraction of records at multiple data sources in the community. 2. Review of all abstracted evaluations by trained study personnel to determine ASD case status. |
| Delobel-Ayoub *et al.* (2020) EU/FR | Yes | 7 – 9y | South West : 84.1%  South East : 79.9% | Regional registries (2007) | ICD-10-CM codes F84.0, F84.1, F84.5, F84.8, F84.9 |
| Doshi-Velez *et al.* (2017) US | No | 0 – 15y | Subgroup 1: 60%  Subgroup 2: 70%  Subgroup 3: 80% Subgroup 4: 80% | National Center for  Biomedical Computing at Boston Children’s Hospital | ICD-9-CM codes 299.00, 299.01, 299.80, 299.81, 299.90, 299.91 |
| Dovgan *et al.* (2019) US | No | 10.1y | 80.6% | National Survey of Children with Special Health Care Needs | Questionnaire |
| Elrod *et al.* (2016) US | Yes | 0 - 17.5y | 80% | TRICARE military health system database | ICD-9-CM codes 299.8, 299.81, 299.0, 299.80,  299.01, or 299.90 |
| Ewen *et al.* (2019) US | No | CAQ cohort: M=10.3y (SD= 3.14)  BDQ cohort: M= 11.9 (SD= 3.21) | CAQ cohort: 83.2%  BDQ cohort: 80.3% | Interactive autism network database | Review of parent and professional-provided medical records |
| Flor *et al.* (2017) US | No | 2 – 4y ; 5 – 7y ; 8 – 11y  12 – 14y ; 15 – 17y | 83.4% | ATN registry | Children: Multidisciplinary autism evaluations including medical evaluation, comprehensive, diagnostic interview, cognitive measure, adaptive skills assessment, ADOS and ATN forms for  Caretakers: ATN forms and questionnaires related to sensory processing problems, quality  of life, behavior problems, sleep issues |
| Fulceri *et al.* (2016) ITA | Yes | 3.8y | 82.6% | IRCCS Stella Maris Foundation (Pisa, Italy) | Evaluation by a multidisciplinary team and confirmed by the administration of the ADOS-G. Evaluation was completed audiometry, evaluation of thyroid function, high-resolution karyotyping, DNA analysis of FRA-X and screening tests for inborn errors of metabolism |
| Goldin *et al.* (2014) US | No | M= 8.2y (SD= 4.4)  Range: 3 -16y | ASD: 81.32%  ASD+ID: 83.78 % | Study sample described in Matson, Gonzales, Wilkins, & Rivet, 2008 | Evaluation included a comprehensive assessment battery with structured interviews, ratings scales, behavioral observation, and developmental/medical history. Additionally, all participants met ASD diagnosis criteria according to the DSMIV-TR/ICD-10 Checklist |
| Guinchat *et al.* (2015) FR | No | M= 15.66y (SD=4.07)  Range: 11-33y | 76% | University hospital (Pitié-Salpêtrière) | - CARS score: M= 40.18 (SD=4.76) - ADI-R (mean ; SD):   - Social impairment score: 24.3 (8.2)   - Communication score: 17.3 (5.3)   - Repetitive interest score: 6.5 (2.5)   - Developmental score: 3.83 (1.47) - VABS (mean ; SD):   - Developmental age: 20.7 months (5.9) |
| Houghton *et al.* (2017) US | Yes | 3 – 4y 5 – 11y 12 – 17y | Commercial= 80%  Medicaid= 79% | Truven Health MarketScanVR Commercial Database | ICD-9-CM: 299.0x, 299.8x, 299.9x  Most prevalent ASD diagnosis (Commercial/Medicaid)   - Autistic disorder: 65.16% / 76.14% - Other PDD: 29.72% / 20.49% - Unspecified PDD: 5.12% / 3.37% |
| Houghton *et al.* (2018) UK | Yes | 3 – 4y 5 – 11y 12 – 17y | 81% | CPRD database | READ codes :  E140.12, Eu84500, Eu84011, E140.00, Eu84000, Eu84.00, Eu84100, E140.13, E140000, Eu84z00, E140.11, E140100, E140z00, Eu84012, Eu84112, Eu84y00, Eu84z11, E141.00, E141.11, E141100, Eu84013, Eu84014, Eu84300, Eu84311, Eu84312, Eu84313, Eu84400, Eu84111, Eu84314, Eu84511 |
| Kang *et al.* (2014) US | Yes | 2 – 5y 6 – 12y 13 – 18y | 78.5% | New Jersey Neurosciences Institute | NR |
| Kerns *et al.* (2014) US | No | M= 10.56y (SD= 2.75)  Range: 7 – 17y | 78% | Center for Autism Research of Children’s (Hospital of Philadelphia) | - ADI-R - ADOS-G - SRS - BRBS - SSRS - BASC2 - PSI |
| Kerns *et al.* (2017) US | No | M= 11.50y (SD= 3.50) | 51% | NSCH 2011-2012 | ASD status based on parent report |
| Llanes *et al.* (2018) US | Yes | M= 5.00y (SD= 1.00)  Range: 4-7y | 82% | Two study sites in Southern California and Massachusetts | - ADI-R - ADOS-G |
| Maenner *et al.* (2020) US | Yes | 8y | 80% | ADDM network (2014) | Case determination is a two-phase process:   1. Review and abstraction of records at multiple data sources in the community. 2. Review of all abstracted evaluations by trained study personnel to determine ASD case status. |
| Mansour *et al.* (2017) US | Yes | M= 9.37y (SD= 1.80)  Range: 6.7-13.5y | 79% | Population study from Pearson *et al.* 2012 & Pearson *et al.* 2013 | - SCQ ≥15 - ADI-R - ADOS - Clinical interview |
| Mosner *et al.* (2019) US | Yes | M= 14.03y (SD= 1.95) | 83% | UNC Autism Research Registry (Carolina Institute for Developmental Disabilities) | - ADOS-2 CSS: M=8.60 (SD= 1.34) - SRS-SR / SRS-CR:   - SRS-2 total t score: M=80.06 (SD=9.00) |
| Muratori *et al.* (2019) ITA | Yes | M= 44.01mo (SD= 13.76)  Range: 16.0-75.2mo | 83% | Three Italian care centers (Bambin Gesù Childrne’s Hospital (Pisa), IRCCS Fondazione Stella Maris (Rome), Stella Maris Mediterraneo Foundation (Matera)) | - ADOS-CSS (Social affect): M=6.11 (SD=1.96) - ADOS-CSS (RRB): M=6.95 (SD=0.06) - ADOS-CSS (Total score): M=6.26 (SD=0.06) |
| Musser *et al.* (2014) US | No | Range: 6 -12y | NR | Kaiser Permanente Northwest | ICD-9-CM codes (at least 2 records) 299.00, 299.80, 299.81 |
| Polyak *et al.* (2015) US/CAN | No | NR | NR | Signature Genomic Laboratoires | NR |
| Rydzewska *et al.* (2018) UK | Yes | 0 – 15y  16 – 24y | 79% | Scotland Census 2011 | Self-reporting |
| Salazar *et al.* (2015) UK | Yes | Range: 4 – 8y | 56% | Primary care services | - SCQ   - Study site 1 : M=19.6 (SD=7.2)   - Study site 2 : M= 23.7 (SD= 6.9) - PAPA |
| Skewer *et al.* (2019) US | Yes | Child: M= 7.59 (SD= 1.99)  Adolescent: M= 14.79 (SD= 1.9) | Child: 82%  Adolescent: 69% | Boston University Research center | - Child   - ADI-R (social interaction) : M= 25.94 (SD=2.72)   - ADI-R (nonverbal communication) : M= 11.9 (SD=2.22)   - ADI-R (repetitive behavior) : M= 5.35 (SD=1.49)   - ADOS (social affect): M= 7.12 (SD=1.19)   - ADOS (RRB): M= 8.91 (SD=1.18)   - ADOS (Total CSS): M= 7.70 (SD=1.18) - Adolescent   - ADI-R (social interaction) : M= 26.04 (SD=3.8)   - ADI-R (nonverbal communication) : M= 12.59 (SD=1.53)   - ADI-R (repetitive behavior) : M= 5.96 (SD=2.47)   - ADOS (social affect): M= 7.47 (SD=1.74)   - ADOS (RRB): M= 7.94 (SD=1.70)   - ADOS (Total CSS): M= 7.59 (SD=1.74) |
| Soke *et al.* (2018) US | Yes | 4y ; 8y | 4y= 78% 8y= 31% | ADDM network | Case determination is a two-phase process:   1. Review and abstraction of records at multiple data sources in the community. 2. Review of all abstracted evaluations by trained study personnel to determine ASD case status. |
| Stacy *et al.* (2014) US | No | Male: M= 9.71 (SD= 0.27) Female: M= 10.82 (SD= 0.61) | 82% | National Survey of Children’s Health (2007) | Self-reporting |
| Supekar *et al.* (2017) US | Yes | 0-18y | NR | STRIDE Cohort Discovery Tool | ICD-9-CM codes, CPT codes |
| Thomas *et al.* (2016) US | Yes | M= 4.2y (SD= 2.7) | 82% | The National Survey of Children’s Health | Self-reporting |
| Vargason *et al.* (2019) US | No | 5 – 15y | 82% | OptumLabs^®^ Data Warehouse | ICD-9-CM codes 299.0x, 299.8x, 299.9x, 299.1x, 330.8x |
| Viscidi *et al.* (2014) US | No | M= 9y (SD= 3.6y) | NR | SSC | - ADOS - ADI-R |

*ADDM= Autism and Developmental Disabilities Monitoring ; ADI-R= Autism Diagnostic Interview-Revised ; ADOS-CSS= Autism Diagnostic Observation Schedule Calibrated Severity Score ; ADOS-G= Autism Diagnostic Observation Schedule-Generic ; AGRE= Autism Genetics Research Exchange ; BASC2= Behavior Assessment Scale for Children, Second Edition-Parent Scale ; BDQ= Birth and ASD Diagnosis Questionnaire ; ATN= Autism Treatment Network ; CAQ= Child with ASD Questionnaire ; CARS= Childhood Autism Rating Scale ; CPRD= Clinical Practice Research Datalink ; CPT= Current Procedural Terminology ; DSM= Diagnostic and Statistical Manual of mental disorders ; ECBI= Eyberg Child Behavior Inventory ; ICD-9-CM= International Classification of Diseases, 9^th^ Edition, Clinical Modification ; ID= Intellectual Disability ; IRCCS= Istituto di Ricovero e Cura a Carattere Scientifico ; M= Mean ; NSCH= National Survey of Children's Health ; PAPA= Preschool Age Psychiatric Assessment ; PDD= Pervasive Developmental Disorder ; PSI= Parenting Stress Index ; RBS= Repetitive Behavior Scale ; RRB= Restricted Repetitive Behavior ; SD= Standard Deviation ; SRS= Social Responsiveness Scale ; SRS-CR= Social Responsiveness Scale Caregiver-Report ; SRS-SR= Social Responsiveness Scale Self-Report ; SSC= Simons Simplex Collection ; SSRS= Social Skill Rating System ; STRIDE= Standford Translational Research Integrated Database Environment ; TRF= Teacher Report Form ; UNC= University of North Carolina ; VABS= Vineland Adaptive Behavior Scales*

Supplement 5: Prevalence of co-morbidities in ASD – Summary of results

|  |  | **Study age group**  **(Range or Mean (SD))** | **Age group** | | |
| --- | --- | --- | --- | --- | --- |
|  |  |  | **0 - 5y** | **6 - 11y** | **12 - 17y** |
| **ADHD** | *Brookman-Frazee et al. (2018)* | 4 - 14y | 78.00% | | |
|  | *Guinchat et al. (2015)* | 11 - 33y | - | 1.70% | |
|  | *Houghton et al. (2017)* | 3 - 4y ; 5 - 11y ;12 - 17y | 6.89% - 47.67% | | 47.73% - 51.08% |
|  | *Houghton et al. (2018)* | 3 - 4y ; 5 - 11y ; 12 - 17y | 0.00% - 10.40% | | 17.70% |
|  | *Kerns et al. (2014)* | 7 - 17y | - | 41.00% | |
|  | *Kerns et al. (2017)* | 11.50y (3.50) | - | 49.00% | |
|  | *Llanes et al. (2018)* | 4 - 7y | 20.30% - 44.70% | | |
|  | *Mansour et al. (2017)* | 6.7 - 13.5y | 86% | | |
|  | *Mosner et al. (2019)* | 14.03y (1.95) | - | - | 71% |
|  | *Muratori et al. (2019)* | 16.0 - 75.2mo | 17.30% | - | - |
|  | *Musser et al. (2014)* | 6 - 12y | 0.20% | | - |
|  | *Polyak et al. (2015)* | N/A | 3.00% - 16.00% | | |
|  | *Salazar et al. (2015)* | 4 - 8y | 59.10%  (Male: 64.40% ; Female: 38.60%) | | - |
|  | *Skwerer et al. (2019)* | Child: 7.49 (1.99) Adolescent: 14.79 (1.9) | 18.83% - 48.50% | | 18.75% - 31.25% |
|  | *Soke et al. (2018)* | 4y ; 8y | 5.49% | 26.05% | - |
|  | *Stacy et al. (2013)* | Male: 9.71 (0.27) Female: 10.82 (0.61) | - | Male: 12.80% - 30.60% Female: 15.60% - 20.30% | - |
|  | *Supekar et al. (2017)* | 0 - 18y | Male: 46.39% Female: 31.25% | | |
| **Anxiety** | *Brookman-Frazee et al. (2018)* | 4 - 14y | 56.00% | | |
|  | *Houghton et al. (2017)* | 3 - 4y ; 5 - 11y ;12 - 17y | 2.59% - 18.87% | | 17.73% - 30.49% |
|  | *Houghton et al. (2018)* | 3 - 4y ; 5 - 11y ; 12 - 17y | 0.00% - 1.70% | | 4.80% |
|  | *Kerns et al. (2014)* | 7 - 17y | - | 48.00% | |
|  | *Kerns et al. (2017)* | 11.50 (3.50) | - | 40.50% | |
|  | *Llanes et al. (2018)* | 4 - 7y | 4.70% - 50.00% | | |
|  | *Mansour et al. (2017)* | 6.7 - 13.5y | 1.00% - 6.00% | | |
|  | *Mosner et al. (2019)* | 14.03y (1.95) | - | - | 34.30% |
|  | *Muratori et al. (2019)* | 16.0 - 75.2mo | 16.70% | | - |
|  | *Salazar et al. (2015)* | 4 - 8y | 78.90%  (Male: 82.20% ; Female: 65.90%) | |  |
|  | *Skwerer et al. (2019)* | Child: 7.49 (1.99) Adolescent: 14.79 (1.9) | - | 0.00% - 12.12% | 3.13% - 6.20% |
|  | *Soke et al. (2018)* | 4y ; 8y | 4.73% | 11.18% |  |
|  | *Stacy et al. (2013)* | Male: 9.71 (0.27) Female: 10.82 (0.61) | - | Male: 11.10% - 24.90% Female: 11.70% - 18.60% | - |
| **Depressive disorder** | *Brookman-Frazee et al. (2018)* | 4 - 14y | 30.00% | | |
|  | *Houghton et al. (2017)* | 3 - 4y ; 5 - 11y ;12 - 17y | 0.44% - 5.08% | | 12.31% - 13.11% |
|  | *Houghton et al. (2018)* | 3 - 4y ; 5 - 11y ; 12 - 17y | 0.00% - 0.90% | | |
|  | *Kerns et al. (2014)* | 7 - 17y | - | 5% | |
|  | *Kerns et al. (2017)* | 11.50 (3.50) | - | 20.90% | |
|  | *Mansour et al. (2017)* | 6.7 - 13.5y | 1.00% | | |
|  | *Mosner et al. (2019)* | 14.03y (1.95) | - | - | 28.60% |
|  | *Muratori et al. (2019)* | 16.0 - 75.2mo | 23.40% | | - |
|  | *Salazar et al. (2015)* | 4 - 8y | 14.60%  (Male: 17.20% ; Female: 4.50%) | | - |
|  | *Skwerer et al. (2019)* | Child: 7.49 (1.99) Adolescent: 14.79 (1.9) | 0.00% | | 3.13% |
|  | *Soke et al. (2018)* | 4y ; 8y | 56.00% | 74.80% | - |
|  | *Stacy et al. (2013)* | Male: 9.71 (0.27) Female: 10.82 (0.61) | - | Male: 4.50% - 7.80% Female: 3.60% - 7.20% | - |
| **Epilepsy** | *Aldinger et al. (2015)* | 3 cohorts: 9.2y (5.1) / 9.3y (5.2) / 9.0y (3.6) | 5.30% - 12.20% | | |
|  | *Doshi-Velez et al. (2014)* | 0 - 15y | 18.16% - 77.50% | | |
|  | *Ewen et al. (2019)* | 2 cohorts: 10.3y (3.14) / 11.9y (3.21) | - | 9.10% - 10.90% | |
|  | *Guinchat et al. (2015)* | 11 - 33y | - | 20.69% - 38.00% | |
|  | *Houghton et al. 2017* | 3 - 4y ; 5 - 11y ;12 - 17y | 3.82% - 7.40% | | 2.80% |
|  | *Houghton et al. 2018* | 3 - 4y ; 5 - 11y ; 12 - 17y | 2.80% | | 4.10% |
|  | *Soke et al. (2018)* | 4y ; 8y | 2.81% | 3.02% | - |
|  | *Stacy et al. (2013)* | Male: 9.71 (0.27) Female: 10.82 (0.61) | - | Male: 3.20% - 7.40% Female: 4.60% - 5.10% | - |
|  | *Supekar et al. (2017)* | 0 - 18y | Male: 40.36%  Female: 43.75% | | |
|  | *Thomas et al. (2017)* | 4.2y (2.7) | 8.60% | - | - |
|  | *Vargason et al. (2019)* | 5 - 15y | 17.40% | | |
|  | *Viscidi et al. (2014)* | 9y (3.6) | 2.20% | | |
| **Gastro-Intestinal disorders** | *Aldinger et al. (2015)* | 3 cohorts: 9.2y (5.1) / 9.3y (5.2) / 9.0y (3.6) | 42.20% - 44.50% | | |
|  | *Doshi-Velez et al. (2014)* | 0 - 15y | 3.43% - 24.37% | | |
|  | *Flor et al. (2017)* | 2 – 4y ; 5 – 7y ; 8 – 11y ; 12 – 14y ; 15 – 17y | 7.00% - 33.30% | | |
|  | *Fulceri et al. (2016)* | 3.8y | 37.40%  (Male: 29.60% ; Female: 7.80%) | - | - |
|  | *Kang et al. (2014)* | 2 – 5y ; 6 – 12y ; 13 – 18y | 49.00% | | 44.00% |
|  | *Supekar et al. (2017)* | 0 - 18y | Male: 0.60% - 25.90%  Female: 0.00% - 18.75% | | |
|  | *Vargason et al. (2019)* | 5 - 15y | 67.80% | | |
| **Hearing Impairment** | *Polyak et al. (2015)* | N/A | Male: 0.00% - 2.18% Female: 0.00% - 0.10% | | |
|  | *Stacy et al. (2013)* | Male: 9.71 (0.27) Female: 10.82 (0.61) | - | Male: 2.00% - 4.20% Female: 2.50% - 4.80% | - |
|  | *Rydzewska et al. (2013)* | 0 – 15y ; 16 – 24y | 2.90%  (Male: 2.40% ; Female: 4.90%) | | |
| **Intellectual Disability** | *Christensen et al. (2019)* | 4y | 46.10% |  |  |
|  | *Delobel-Ayoub et al. (2019)* | 7 - 9y | - | 24.00% - 38.90% | - |
|  | *Doshi-Velez et al. (2014)* | 0 - 15y | 12.70% - 60.00% | | |
|  | *Dovgan et al. (2019)* | 10.1y | - | 79.00% - 91.70% | - |
|  | *Goldin et al. (2014)* | 3 - 16y | 16.89% | | |
|  | *Guinchat et al. (2015)* | 11 - 33y | - | 12.00% - 47.00% | - |
|  | *Houghton et al. (2017)* | 3 - 4y ; 5 - 11y ;12 - 17y | 0.81% - 15.19% | | 4.66% - 20.58% |
|  | *Houghton et al. (2018)* | 3 - 4y ; 5 - 11y ; 12 - 17y | 0.00% - 0.40% | | |
|  | *Llanes et al. (2015)* | 4 - 7y | 17.80% | | - |
|  | *Maenner et al. (2020)* | 8y | - | 33.00% | - |
|  | *Polyak et al. (2015)* | N/A | Male: 21.00% - 23.00% Female: 22.00% - 26.00% | | |
|  | *Rydzewska et al. (2018)* | 0 – 15y ; 16 – 24y | 13.60%  (Male: 11.3% ; Female: 22.80%) | | |
|  | *Thomas et al. (2017)* | 4.2y (2.7) | 22.30% | - | - |
| **Sleep Disorders** | *Aldinger et al. (2015)* | 3 cohorts: 9.2y (5.1) / 9.3y (5.2) / 9.0y (3.6) | 55.00% - 72.50% | | |
|  | *Elrod et al. (2016)* | 0 - 17.5y | 31.00% | | |
|  | *Houghton et al. (2017)* | 3 - 4y ; 5 - 11y ;12 - 17y | 5.87% - 12.79% | | 5.14% - 8.85% |
|  | *Houghton et al. (2018)* | 3 - 4y ; 5 - 11y ; 12 - 17y | 6.40% - 13.20% | | 14.40% |
|  | *Soke et al. (2013)* | 4y ; 8y | 26.95% | 37.12% | - |
|  | *Supekar et al. (2017)* | 0 - 18y | Male: 3.01% Female: 2.08% | | |
|  | *Vargason et al. (2019)* | 5 - 15y | 20.00% | | |
| **Vision Impairment** | *Aldinger et al. (2015)* | 3 cohorts: 9.2y (5.1) / 9.3y (5.2) / 9.0y (3.6) | 14.90% - 15.30% | | |
|  | *Polyak et al. (2015)* | N/A | Male: 0.00%  Female: 0.00% - 0.10% | | |
|  | *Rydzewska et al. (2018)* | 0 – 15y ; 16 – 24y | 3.50% | | |

*N/A= Not Available ; SD= Standard Deviation*

Supplement 6: Prevalence of co-morbidities in ASD – Main results

| **Author (Year)** | **Method to estimate prevalence** | **ASD cases** | **Prevalence estimate** |
| --- | --- | --- | --- |
| **ADHD (n=17 studies)** |  |  |  |
| Brookman-Frazee *et al.* (2018) | MINI-KID-P | 201 | Any ADHD disorder 78.0%  Hyperactive/impulsive= 4.00% Inattentive = 11.00% Combined= 62.00% |
| Guinchat *et al.* (2015) | Checklist based on Perisse *et al.* 2010 | 58 | 1.70% (n=1)* |
| Houghton *et al.* (2017) | ICD-9-CM code 314x | Commercial= 46,943 Medicaid= 46,696 | Commercial: 3 – 4y= 6.89% 5 – 11y= 40.46% 12 – 17y= 47.73%  Medicaid: 3 – 4y= 14.19% 5 – 11y= 47.67% 12 – 17y= 51.08% |
| Houghton *et al.* (2018) | READ codes 6A61.00, 9Ol8.00, 9OlA.00, E2E..00, E2E0.00, E2E0100, Eu90000, Eu90011, ZS91.00, ZS91.11, ZS91.12 | 10,856 | 3 – 4y= 0.00% (n=0) 5 – 11y= 10.40% (n=293) 12 – 17y= 17.70% (n=598) |
| Kerns *et al.* (2014) | DSM-IV-TR | NR | 41.00% |
| Kerns *et al.* (2017) | Review of parent reports | 1,280 | ADD/ADHD= 49.0% |
| Llanes *et al.* (2018) | CBCL and TRF (parent and teacher reports) | 180 | Parents: ≤5y= 22.10% (n=19) ≥6y= 44.70% (n=42)  Teachers: ≤5y= 20.30% (n=13) ≥6y= 28.80% (n=19) |
| Mansour *et al.* (2017) | DICA-IV CPRS-R:L CTRS-R:L | 99 | ADHD : 86.00% (n=85)  Hyperactive/impulsive= 21.00% Inattentive = 1.00% Combined= 64.00% |
| Mosner *et al.* (2019) | MINI-KID-P | 35 | 71.00% (n=25) |
| Muratori *et al.* (2019) | CBCL1^1/2^-5 (Italian version) | 989 | 17.30% (n=171) |
| Musser *et al.* (2014) | ICD-9-CM codes 314.00, 314.01 | 297 | 0.20% (n=64) |
| Polyak *et al.* (2015) | Review of medical records | 3,602 | Clinical: Male= 0.12 Female= 0.09  Rare CNV: Male= 0.16 Female= 0.03 |
| Salazar *et al.* (2015) | PAPA (electronic version) | 101 | All= 59.10% (CI_95%_: 47.30% - 70.90%)  Male= 64.40% (CI_95%_: 50.00% - 78.70%)  Female= 38.60% (CI_95%_: 23.90% - 53.40%) |
| Skwerer *et al.* (2019) | CASI-5 | Child: 33  Adolescent: 32 | All= 18.50% - 40.00%  Hyperactive/impulsive= 23.1%  Inattentive = 40%  Combined= 18.5%  Child= 18.83% - 48.50%  Hyperactive/impulsive= 21.21%  Inattentive = 48.5%  Combined= 18.18  Adolescent= 18.75% - 31.25%  Hyperactive/impulsive= 25%  Inattentive = 31.25%  Combined= 18.75% |
| Soke *et al.* (2018) | Review of medical records | 4y= 607  8y=1,091 | 4y= 5.49% (n=43)  8y= 26.05% (n=306) |
| Stacy *et al.* (2013) | Phone interview | Male: 746 Female= 167 | Male: Mild= 12.80% Moderate/severe= 30.60%  Female: Mild= 15.60% Moderate/severe= 20.30% |
| Supekar *et al.* (2017) | ICD-9-CM | 4,790 | Male= 46.39% Female= 31.25% |
| **Anxiety (n=13 studies)** |  |  |  |
| Brookman-Frazee *et al.* (2018) | MINI-KID-P | 201 | All: 56.00%  Social phobia: 24%  Separation anxiety disorder:15% |
| Houghton *et al.* (2017) | ICD-9-CM codes 300.00-300.29, 301.4x | Commercial= 46,943 Medicaid= 46,696 | Commercial: 3 – 4y= 3.65% 5 – 11y= 18.87% 12 – 17y= 30.49%  Medicaid: 3 – 4y= 2.59% 5 – 11y= 11.50% 12 – 17y= 17.73% |
| Houghton *et al.* (2018) | READ codes E200.00, E200z00, E200200, Eu41100, E200400, Eu41.00, Eu41000, Eu41300, Eu41y00, Eu41z00, E200000, E200100, E200500 | 10,856 | 3 – 4y= 0.00% (n=0) 5 – 11y= 1.70% (n=48) 12 – 17y= 4.80% (n=162) |
| Kerns *et al.* (2014) | DSM-IV-TR | NR | 48.00% |
| Kerns *et al.* (2017) | Review of parent reports | 1,280 | 40.50% (n=613) |
| Llanes *et al.* (2018) | CBCL and TRF (parent and teacher reports) | 180 | Parents: ≤5y= 31.40% (n=27) ≥6y= 50.00% (n=47)  Teachers: ≤5y= 4.70% (n=3) ≥6y= 30.70% (n=24) |
| Mansour *et al.* (2017) | DICA-IV CPRS-R:L CTRS-R:L | 99 | Generalized anxiety disorder= 2.00% (n=2)  Social phobia: 6% (n=6)  Separation anxiety disorder= 1.00% (n=85) |
| Mosner *et al.* (2019) | MINI-KID-P | 35 | 34.30% (n=12) |
| Muratori *et al.* (2019) | CBCL1^1/2^-5 (Italian version) | 989 | 16.70% (n=165) |
| Salazar *et al.* (2015) | PAPA (electronic version) | 101 | All= 78.90% (CI_95%_: 70.70% - 87.00%)  Male= 82.20% (CI_95%_: 72.60% - 91.80%)  Female= 65.90% (CI_95%_: 51.50% - 80.30%) |
| Skwerer *et al.* (2019) | CASI-5 | Child: 33  Adolescent: 32 | All= 1.5% - 9.2%  Generalized anxiety disorder= 3.1%  Social phobia: 9.2%  Separation anxiety disorder= 3.1%  Child= 0% - 12.12%  Generalized anxiety disorder= 3.03%  Social phobia: 12.12%  Separation anxiety disorder= 3.03%  Adolescent= 3.13% - 6.2%  Generalized anxiety disorder= 3.13%  Social phobia: 6.2%  Separation anxiety disorder= 3.03% |
| Soke *et al.* (2018) | Review of medical records | 4y= 607  8y=1,091 | 4y= 4.73% (n=37)  8y= 11.18% (n=122) |
| Stacy *et al.* (2013) | Phone interview | Male: 746 Female= 167 | Male: Mild= 11.10% Moderate/severe= 24.90%  Female: Mild=11.70% Moderate/severe= 18.60% |
| **Depressive disorders (n=12 studies)** |  |  |  |
| Brookman-Frazee *et al.* (2018) | MINI-KID-P | 201 | 30.00% (n=60) (mood disorders) |
| Houghton *et al.* (2017) | ICD-9-CM codes 296.20-296.35, 311x | Commercial= 46,943 Medicaid= 46,696 | Commercial: 3 – 4y= 0.44% 5 – 11y= 2.42% 12 – 17y= 13.11%  Medicaid: 3 – 4y= 3.28% 5 – 11y= 5.08% 12 – 17y= 12.31% |
| Houghton *et al.* (2018) | READ codes Eu32.00, Eu32.11-13, Eu32000, Eu32100, Eu32200, Eu32211-13, Eu32300, Eu32311-14, Eu32400, Eu32500, Eu32600, Eu32700, Eu32800, Eu32y00, Eu32y11-12, Eu32z00, Eu32z11-14, Eu33.00, Eu33.14-15, Eu33000, Eu33100, Eu33200, Eu33214, Eu33211, Eu33311, Eu33400, Eu33y00, Eu33z00, Eu33z11, Eu34100, Eu34114, Eu41200, Eu41211, Eu53011, Eu53012, E118.00, E135.00, E2B..00, E2B1.00, E291.00, E204.00, E204.11, E2B0.00, E112.00, E112.11-14, E112000, E112100, E112200, E112300, E112500, E112600, E112z00, E113.00, E113000, E113100, E113200, E113300, E113500, E113600, E113700, E113z00, E11z200, E11y200, E200300, E02y300 | 10,856 | 3 – 4y= 0.00%  5 – 17y= 0.90% (n=54) |
| Kerns *et al.* (2014) | DSM-IV-TR | NR | 5.00% |
| Kerns *et al.* (2017) | Review of parent reports | 1,280 | 20.90% (n=284) |
| Mansour *et al.* (2017) | DICA-IV CPRS-R:L CTRS-R:L | 99 | Major depressive disorder= 1.00% (n=1) |
| Mosner *et al.* (2019) | MINI-KID-P | 35 | 28.60% (n=10)  Past episode= 14.30% (n=5) Recurrent= 14.30% (n=5) |
| Muratori *et al.* (2019) | CBCL1^1/2^-5 (Italian version) | 989 | 23.40% (n=231) |
| Salazar *et al.* (2015) | PAPA (electronic version) | 101 | All= 14.60% (major depression) (CI_95%_: 6.00% - 23.20%)  Male= 17.20% (CI_95%_: 6.40% - 27.90%)  Female= 4.5% (CI_95%_: 0.0% - 10.9%) |
| Skwerer *et al.* (2019) | CASI-5 and EDI | Child: 33  Adolescent: 32 | Major depressive disorder  All= 1.5%  Child= 0%  Adolescent= 3.13% |
| Soke *et al.* (2018) | Review of medical records | 4y= 607  8y=1,091 | 4y= 56.00% (n=438)  8y= 74.80% (n=816) |
| Stacy *et al.* (2013) | Phone interview | Male: 746 Female: 167 | Male: Mild= 4.50% Moderate/severe= 7.80%  Female: Mild= 7.20% Moderate/severe= 3.60% |
| **Epilepsy/Seizure (n=12 studies)** |  |  |  |
| Aldinger *et al.* (2015) | ADI-R | AGRE Cohort:  Multiplex cohort: 728  Cluster analysis sample: 627  SSC Cohort: 2,623 | AGRE cohort (multiplex)= 12.20%  AGRE cohort (Cluster analysis sample)= 11.20%    SSC Cohort= 5.30% |
| Doshi-Velez *et al.* (2014) | ICD-9-CM | Subgroup 1: 120  Subgroup 2: 197  Subgroup 3: 212  Subgroup 4: 4,316 | Subgroup 1= 77.50% (CI_95%_: 70.03% - 84.97%)  Subgroup 2= 42.13% (CI_95%_: 35.24% - 49.03%)  Subgroup 3= 33.02% (CI_95%_: 26.69% - 39.35%)  Subgroup 4= 18.16% (CI_95%_: 17.01% - 19.32%) |
| Ewen *et al.* (2019) | CAQ and BDQ questionnaires | CAQ : 4,801  BDQ: 1,736 | CAQ cohort= 9.10%  BDQ cohort= 10.90% |
| Guinchat *et al.* (2015) | Checklist based on Perisse *et al.* 2010, electroencephalography | 58 | Seizures= 20.69% (n=12)* Epileptic encephalopathy= 38.00% (n=22) |
| Houghton *et al.* 2017 | ICD-9-CM code 345x | Commercial= 46,943 Medicaid= 46,696 | Commercial: 3 – 4y= 3.82% 5 – 11y= 5.21% 12 – 17y= 7.04%  Medicaid: 3 – 4y= 5.41% 5 – 11y= 7.40% 12 – 17y= 10.38% |
| Houghton *et al.* 2018 | READ codes F132100, F25..00, F250.00, F250000, F250011, F250200, F250300, F250500, F250y00, F250z00, F251.00, F251000, F251011, F251200, F251300, F251400, F251500, F251y00, F251z00, F252.00, F253.00, F253.11, F254.00, F254000, F254100, F254200, F254300, F254400, F254500, F254z00, F255.00, F255000 F255011, F255012, F255100, F255200, F255300, F255311, F255400, F255500, F255600, F255y00, F255z00, F257.00, F25B.00, F25C.00, F25D.00, F25E.00, F25F.00, F25X.00, F25y.00, F25y000, F25y100, F25y200, F25y300, F25y500, F25yz00, F25z.00, F25z.11, SC20000 | 10,856 | 3 – 11y= 2.8% (n=83) 12 – 17y= 4.1% (n=137) |
| Soke *et al.* (2018) | Review of medical records | 4y= 607  8y=1,091 | 4y= 2.81% (n=22)  8y= 3.02% (n=33) |
| Stacy *et al.* (2013) | Phone interview | Male: 746 Female= 167 | Male: Mild= 7.40% Moderate/severe= 3.20%  Female: Mild= 5.10% Moderate/severe= 4.60% |
| Supekar *et al.* (2017) | ICD-9-CM | 4,790 | Male= 40.36% Female= 43.75% |
| Thomas *et al.* (2017) | Telephone survey | 1,604 | 8.60% |
| Vargason *et al.* (2019) | ICD-9-CM codes 555.xx, 556.xx, 558.xx, 564.0x, 564.1x, 787.91, 789.0x | 3,278 | 17.4% |
| Viscidi *et al.* (2014) | ADI-R | 2,645 | 2.2% |
| **Gastro-Intestinal Syndromes (n=7 studies)** |  |  |  |
| Aldinger *et al.* (2015) | Method reported in Campbell *et al.* (2009) | AGRE Cohort:  Multiplex cohort: 728  Cluster analysis sample: 627  SSC Cohort: 2,623 | AGRE cohort (multiplex)= 42.40%  AGRE cohort (Cluster analysis sample)= 44.5%    SSC Cohort= 43.1% |
| Doshi-Velez *et al.* (2014) | ICD-9-CM | Subgroup 1: 120  Subgroup 2: 197  Subgroup 3: 212  Subgroup 4: 4,316 | Subgroup 1= 14.17% (CI_95%_: 7.93% - 20.41%)  Subgroup 2= 24.37% (CI_95%_: 18.37% - 30.36%)  Subgroup 3= 10.85% (CI_95%_: 6.66% - 15.04%)  Subgroup 4= 3.43% (CI_95%_: 2.89% - 3.97%) |
| Flor *et al.* (2017) | CSHQ | Complex ASD: 75  Essential ASD: 1,272 | Complex ASD : Constipation= 26.70% (n= 20) Other GI problems= 17.30% (n= 13) Either= 33.30% (n= 25)  Essential ASD : Constipation= 13.10% (n= 166) Other GI problems= 7.00% (n= 89) Either= 18.50% (n= 234) |
| Fulceri *et al.* (2016) | CBCL 1^1/2^- 5 | 115 | Total= 37.40% Male= 29.60% Female= 7.80% |
| Kang *et al.* (2014) | Clinical expert assessment | 164 | 49.00% |
| Supekar *et al.* (2017) | ICD-9-CM | 4,790 | Bowel disorders: Male= 25.90% Female= 18.75%  Inflammatory bowel disorders: Male= 0.60% Female= 0.00% |
| Vargason *et al.* (2019) | ICD-9-CM codes 345.xx, 780.39 | 3,278 | 67.8% |
| **Hearing Impairment (n=3 studies)** |  |  |  |
|  |  |  |  |
| Polyak *et al.* (2015) | Review of medical records | 3,602 | Clinical: Male= 0.00218 Female= 0.001  Rare CNV: Male= 0.00 Female= 0.00 |
| Stacy *et al.* (2013) | Phone interview | Male: 746 Female: 167 | Male: Mild= 2.00% Moderate/severe= 4.20%  Female: Mild= 2.50% Moderate/severe= 4.80% |
| Rydzewska *et al.* (2013) | Census survey | 25,063 | 0-15y: Overall= 2.9% Male= 2.4% Female= 4.9% |
|  |  |  |  |
| **Intellectual Disability (n=13 studies)** |  |  |  |
| Christensen *et al.* (2019) | Review of medical records | NR | 46.10% (n= 234) |
| Delobel-Ayoub *et al.* (2019)^¥^ | ICD-10-CM codes F70-79 | South West : 113  South East : 154 | South West : 38.90% (n= 44)  South East : 24.00% (n=37) |
| Doshi-Velez *et al.* (2014) | ICD-9-CM | Subgroup 1: 120  Subgroup 2: 197  Subgroup 3: 212  Subgroup 4: 4,316 | Subgroup 1= 60.00% (CI_95%_: 51.23% - 68.77%)  Subgroup 2= 48.73% (CI_95%_: 41.75% - 55.71%)  Subgroup 3= 27.83% (CI_95%_: 21.80% - 33.86%)  Subgroup 4= 12.70% (CI_95%_: 11.70% - 13.69%) |
| Dovgan *et al.* (2019) | Questionnaire | 3,055 | Autism= 79.00%  Autism (1 co-morbidity)= 85.30%  Autism (2 co-morbidity)= 87.70%  Autism (3 co-morbidity)= 91.70%  Autism (4 co-morbidity)= 85.50% |
| Goldin *et al.* (2014) | WISC-IV and SB5 | 219 | 16.89%* |
| Guinchat *et al.* (2015) | Checklist based on Perisse *et al.* 2010 | 58 | Mild= 12.00% (n=7) Moderate= 16.00% (n=9) Severe= 47.00% (n=27) Profound= 22.00% (n=13) |
| Houghton *et al.* (2017) | ICD-9-CM codes 317x-319x | Commercial= 46,943 Medicaid= 46,696 | Commercial: 3 – 4y= 0.81% 5 – 11y= 2.80% 12 – 17y= 4 .66%  Medicaid: 3 – 4y= 16.66% 5 – 11y= 15.19% 12 – 17y= 20.58% |
| Houghton *et al.* (2018) | READ codes E3...00, E30..00, E30..11, E30..12, E30..13, E31..00, E310.00, E310.11, E311.00, E312.00, E312.11, E31z.00, E3y..00, E3z..00, Eu7..00, Eu70.00, Eu70.11, Eu70.12, Eu70000, Eu70100, Eu70y00, Eu70z00, Eu71.00, Eu71.11, Eu71000, Eu71100, Eu71y00, Eu71z00, Eu72.00, Eu72.11, Eu72000, Eu72100, Eu72y00, Eu72z00, Eu73.00, Eu73.11, Eu73000, Eu73100, Eu73y00, Eu73z00, Eu7y.00, Eu7y000, Eu7y100, Eu7yy00, Eu7yz00, Eu7z.00, Eu7z.11, Eu7z.12, Eu7z000, Eu7z100, Eu7zy00, Eu7zz00 | 10,856 | 3 – 4y= 0.00% (n=0) 5 – 17y= 0.40% (n=22) |
| Llanes *et al.* (2018) | WPPSO-III | 180 | 17.80% |
| Maenner *et al.* (2020) | Review of medical records |  | 33.00% (n=1,059) |
| Polyak *et al.* (2015) | Review of medical records | 3,602 | Clinical: Male= 0.23 Female= 0.26  Rare CNV: Male= 0.21 Female= 0.22 |
| Rydzewska *et al.* (2018) | Census survey | 25,063 | 0-15y: Overall= 13.6% Male= 11.3% Female= 22.8% |
| Thomas *et al.* (2017) | Telephone survey | 1,604 | 22.3% |
| **Sleep Disorders (n=7 studies)** |  |  |  |
| Aldinger *et al.* (2015) | Medical record review | AGRE Cohort:  Multiplex cohort: 728  Cluster analysis sample: 627  SSC Cohort: 2,623 | AGRE cohort (multiplex)= 55.50%  AGRE cohort (Cluster analysis sample)= 55.00%    SSC Cohort= 72.50% |
| Elrod *et al.* (2016) | ICD-9-CM codes 327.53, 780.55, 307.45, 327.39, 307.43, 770.81, 770.82, 307.41, 307.42, 327.02, 327.09, 780.52, 347.00, 347.10, 788.36, 788.30, 307.46, 327.51, 333.94, 327.20, 327.21, 327.23, 327.26, 780.51, 780.53, 780.57, 786.03, 307.40, 307.47, 780.50, 780.58, 780.59, 314.00, 314.01 | 48,762 | 31.00% |
| Houghton *et al.* (2017) | ICD-9-CM code 780.5x | Commercial= 46,943 Medicaid= 46,696 | Commercial: 3 – 4y= 6.39% 5 – 11y= 5.87% 12 – 17y= 5.14%  Medicaid: 3 – 4y= 12.79% 5 – 11y= 10.22% 12 – 17y= 8.85% |
| Houghton *et al.* (2018) | READ codes R005z00, R005900, R005800, R005700, R005600, R005500, R005400, R005312, R005311, R005300, R005200, R005100, R005000, R005.12, R005.11, R005.00, Fy02.00, Fy01.00, Fy00.00, Fy0..00, Eu51200, Eu51100, Eu51000, Eu51.00, E274z00, E274y11, E274y00, E274F00, E274E00, E274D11, E274D00, E274C00, E274B00, E274A00, E274900, E274800, E274700, E274600, E274500, E274400, E274311, E274300, E274200, E274111, E274100, E274000, E274.12, E274.11, E274.00, 1B1Q.00, 1B1B200, 1B1B100, 1B1B000, 1B1B.11, 1B1B.00 | 10,856 | 3 – 4y= 6.40% (n= 7)  5 – 11y= 13.2% (n= 372) 12 – 17y= 14.4% (n= 485) |
| Soke *et al.* (2013) | Review of medical records | 4y= 607  8y=1,091 | 4y= 26.95% (n=211)  8y= 37.12% (n=405) |
| Supekar *et al.* (2017) | ICD-9-CM | 4,790 | Male= 3.01% Female= 2.08% |
| Vargason *et al.* (2019) | ICD-9-CM codes 307.41, 307.42,327.0x, 780.52, 307.43, 307.44,327.1x, 780.54, 307.46, 307.48,327.4x, 780.56, 327.2x, 780.51,780.53,780.57, 786.03, 770.81, 770.82, 780.55, 307.45,327.3x, 327.5x, 333.94,780.58, 307.40, 307.47,307.49, 327.8x, 780.50, 780.59 | 3,278 | 20.00% |
| **Vision Impairment (n=3 studies)** |  |  |  |
| Aldinger *et al.* (2015) | NR | AGRE Cohort:  Multiplex cohor:728  Cluster analysis sample: 627  SSC Cohort: 2623 | AGRE cohort (multiplex)= 14.90%  AGRE cohort (Cluster analysis sample)= 15.30%    SSC Cohort= NR |
| Polyak *et al.* (2015) | Review of medical records | 3,602 | Clinical: Male= 0.00 Female= 0.001  Rare CNV: Male= 0.00 Female= 0.00 |
| Rydzewska *et al.* (2018) | Census survey | 25,063 | 0-15y: Overall= 3.5% Male= 2.6% Female= 6.7% |

**Calculated ^¥^Published online in 2019 and in print in 2020*

*ADHD= Attention Deficit Hyperactivity Disorder ; ADI-R= Autism Diagnostic Interview-Revised ; ASD= Autism Spectrum Disorder ; BDQ= Birth and ASD Diagnosis Questionnaire ; CAQ= Child with ASD Questionnaire ; CASI-5: Child and Adolescent Symptoms Inventory ; CBCL= Child Behavior Checklist-parent Report ; CSHQ= Children’s Sleep Habits Questionnaire ; CNV= Copy Number Variant ; CPRS-R:L= Conners Parent Rating Scale, Revised-Long ; CTRS-R:L= Conners Teacher Rating Scale, Revised-Long ; EDI= Emotion Dysregulation Inventory ; ICD-9-CM= International Classification of Diseases, 9^th^ Edition, Clinical Modification ; MINI-KID-P= Mini-International Neuropsychiatric interview, Parent version ; NR= Not Reported ; NSCH= National Survey of Children’s health ; ODD= Oppositional Defiant Disorder ; PAPA= Preschool Age Psychiatric Assessment ; SB5= Scales-5^th^ Edition ; SD= Standard Deviation ; TRF= Teacher Report Form ; WISC-IV= Wechsler Intelligence Scale for Children-Fourth Edition ; WPPSI-III= Wechsler Preschool and Primary Scale of Intelligence*
